# The functional spectrum of low-frequency coding variation

**DOI:** 10.1186/gb-2011-12-9-r84

**Published:** 2011-09-14

**Authors:** Gabor T Marth, Fuli Yu, Amit R Indap, Kiran Garimella, Simon Gravel, Wen Fung Leong, Chris Tyler-Smith, Matthew Bainbridge, Tom Blackwell, Xiangqun Zheng-Bradley, Yuan Chen, Danny Challis, Laura Clarke, Edward V Ball, Kristian Cibulskis, David N Cooper, Bob Fulton, Chris Hartl, Dan Koboldt, Donna Muzny, Richard Smith, Carrie Sougnez, Chip Stewart, Alistair Ward, Jin Yu, Yali Xue, David Altshuler, Carlos D Bustamante, Andrew G Clark, Mark Daly, Mark DePristo, Paul Flicek, Stacey Gabriel, Elaine Mardis, Aarno Palotie, Richard Gibbs, the 1000 Genomes Project

**Affiliations:** 1 Department of Biology, Boston College, 140 Commonwealth Avenue, Chestnut Hill, MA 02467, USA; 2 Human Genome Sequencing Center, Baylor College of Medicine, One Baylor Plaza, Houston, TX 77030, USA; 3 Population Genomics Program, Broad Institute, 7 Cambridge Center, Cambridge, MA 02142, USA; 4 Department of Genetics, Stanford University, 300 Pasteur Drive, Stanford, CA 94305, USA; 5 The Wellcome Trust Sanger Institute, Wellcome Trust Genome Campus, Hinxton, Cambridge CB10 1SA, UK; 6 School of Public Health, University of Michigan, 1415 Washington Heights, Ann Arbor, MI 48109, USA; 7 European Bioinformatics Institute, Wellcome Trust Genome Campus, Hinxton, Cambridge CB10 1SD, UK; 8 Institute of Medical Genetics, School of Medicine, Cardiff University, Heath Park, Cardiff CF14 4XN, UK; 9 The Genome Institute, Washington University School of Medicine, 4444 Forest Park Avenue, St Louis, MO 63108, USA; 10 Department of Molecular Biology and Genetics, Cornell University, 107 Biotechnology Building, Ithaca, NY 14853, USA

## Abstract

**Background:**

Rare coding variants constitute an important class of human genetic variation, but are underrepresented in current databases that are based on small population samples. Recent studies show that variants altering amino acid sequence and protein function are enriched at low variant allele frequency, 2 to 5%, but because of insufficient sample size it is not clear if the same trend holds for rare variants below 1% allele frequency.

**Results:**

The 1000 Genomes Exon Pilot Project has collected deep-coverage exon-capture data in roughly 1,000 human genes, for nearly 700 samples. Although medical whole-exome projects are currently afoot, this is still the deepest reported sampling of a large number of human genes with next-generation technologies. According to the goals of the 1000 Genomes Project, we created effective informatics pipelines to process and analyze the data, and discovered 12,758 exonic SNPs, 70% of them novel, and 74% below 1% allele frequency in the seven population samples we examined. Our analysis confirms that coding variants below 1% allele frequency show increased population-specificity and are enriched for functional variants.

**Conclusions:**

This study represents a large step toward detecting and interpreting low frequency coding variation, clearly lays out technical steps for effective analysis of DNA capture data, and articulates functional and population properties of this important class of genetic variation.

## Background

The allelic spectrum of variants causing common human diseases has long been a topic of debate [1,2]. Whereas many monogenic diseases are typically caused by extremely rare (<<1%), heterogeneous, and highly penetrant alleles, the genetic basis of common diseases remains largely unexplained [3]. The results of hundreds of genome-wide association scans have demonstrated that common genetic variation accounts for a non-negligible but modest proportion of inherited risk [4,5], leading many to suggest recently that rare variants may contribute substantially to the genetic burden underlying common disease. Data from deep sampling of small numbers of loci have confirmed the population-genetic prediction [6,7] that rare variants constitute the vast majority of polymorphic sites in human populations. Most are absent from current databases [8], which are dominated by sites discovered from smaller population samples, and are consequently biased toward common variants. Analysis of whole exome data from a modest number of samples (*n *= 35) suggests that natural selection is likely to constrain the vast majority of deleterious alleles (at least those that alter amino acid identity and, therefore, possibly protein function) to low frequencies (<1%) under a plethora of evolutionary models for the distribution of fitness effects consistent with patterns of human exomic variation [9]. However, in order to broadly characterize the contribution of rare variants to human genetic variability and to inform medical sequencing projects seeking to identify disease-causing alleles, one must first be able to systematically sample variants below an alternative allele frequency (AF) of 1%.

Recent technical developments have produced a series of new DNA sequencing platforms that can generate hundreds of gigabases of data per instrument run at a rapidly diminishing cost. Innovations in oligonucleotide synthesis have also enabled a series of laboratory methods for targeted enrichment of specific DNA sequences (Figure S1 in Additional file 1). These capture methods can be applied at low cost, and large scale, to analyze the coding regions of genes, where genomic changes that most likely influence gene function can be recognized. Together, these two technologies present the opportunity to obtain full exome sequence for population samples sufficiently large to capture a substantial collection of rare variants.

The 1000 Genomes Exon Pilot (Exon Pilot) Project set out to use capture sequencing to compile a large catalog of coding sequence variants with four goals in mind: (1) to drive the development of capture technologies; (2) to develop tools for effective downstream analysis of targeted capture sequencing data; (3) to better understand the distribution of coding variation across populations; and (4) to assess the functional qualities of coding variants and their allele frequencies, based on the representation of both common (AF > 10%), intermediate (1% < AF < 10%) and low frequency (AF < 1%) sites. To attain these objectives, while simultaneously improving DNA enrichment methods, we targeted approximately 1,000 genes in 800 individuals, from seven populations representing Africa (LWK, YRI), Asia (CHB, CHD, JPT), and Europe (CEU, TSI) in roughly equal proportions (Table 1).

**Table 1:** Samples, read coverage, SNP calls, and nucleotide diversity in the Exon Pilot dataset

Population	YRI	LWK	CHB	CHD	JPT	CEU	TSI	All
Samples	112	108	109	107	105	90	66	697
Technologies	ILL,454	454	ILL,454	ILL,454	ILL,454	ILL,454	ILL	ILL,454
SNPs	5,175	5,459	3,415	3,431	2,900	3,489	3,281	12,758
%dbSNP	53.8	50.1	52.6	50.3	57.9	65.9	65.6	30.36
Ts/Tv	3.56	3.67	3.74	3.64	3.67	3.47	3.53	3.82
Read coverage (first quartile)	18×	19×	18×	30×	20×	20×	20×	19×
Read coverage (median)	27×	25×	22×	36×	26×	43×	57×	29×
Read coverage (mean)	52×	25×	40×	49×	43×	69×	71×	48×
Read coverage (third quartile)	42×	32×	37×	44×	54×	98×	118×	49×
Heterozygosity, all sites	4.42	4.52	3.34	3.35	3.26	3.54	3.5	-
Heterozygosity, four-fold synonymous sites	9.24	9.16	6.6	6.63	6.43	7.12	7.04	-
Heterozygosity, three-fold synonymous sites	5.01	5.41	4.24	4.39	4.6	3.59	3.59	-
Heterozygosity, two-fold synonymous sites	6.04	6.16	4.447	4.42	4.37	4.74	4.68	-
Heterozygosity, non-synonymous sites	2.74	2.86	2.19	2.21	2.12	2.31	2.29	-

Heterozygosity estimates are given in units of 10^-4 ^per base pair. ILL: Illumina; Ts/Tv, transition/transversion ratio.

## Results and discussion

### Data collection and quality control

Four data collection centers, the Baylor College of Medicine (BCM), the Broad Institute (BI), the Wellcome Trust Sanger Institute, and Washington University applied different combinations of solid-phase or liquid-phase capture, and Illumina or 454 sequencing procedures on subsets of the samples (Materials and methods). In order to aggregate the data for a comparison of analytical methods, a set of consensus exon target regions was derived (Materials and methods; Figure S2 in Additional file 1). After filtering out genes that could not be fully tested because of failed capture or low sequence coverage, and samples that showed evidence of cross-contamination, a final sequence data set was assembled that corresponded to a total of 1.43 Mb of exonic sequence (8,279 exons representing 942 genes) in 697 samples (see section 3, 'Data quality control' and Figure S3 in Additional file 1 for details of our quality control procedures). The project was closely coordinated with two related Pilot programs in the ongoing 1000 Genomes Project, the Trio Sequencing Pilot and the Low Coverage Sequencing Pilot, enabling quality control and performance comparisons.

### Data processing and variant analysis

**Figure 1:**
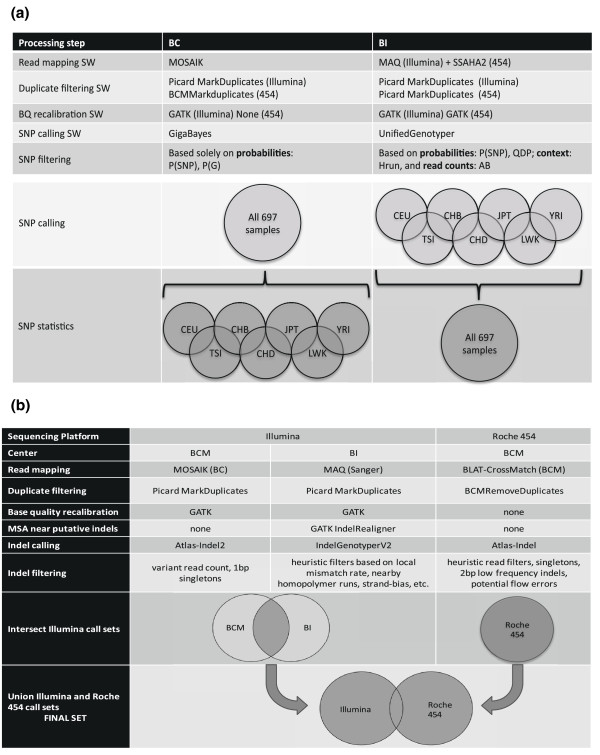
**Variant calling procedure in the Exon Pilot Project**. **(a) **The SNP calling procedure. Read alignment and SNP calling were carried out by Boston College (BC) and the Broad Institute (BI) independently using complementary pipelines. The call sets were intersected for the final release. **(b) **The INDEL calling procedure. INDELs were called on the Illumina and Roche 454 platforms. The sequence was processed on three independent pipelines, Illumina at the Baylor College of Medicine Human Genome Sequencing Center (BCM-HGSC), Illumina at BI, and Roche 454 at BCM-HGSC. The union of the three call sets formed the final call set. The Venn diagram provided is not to scale. AB: allele balance; MSA: multiple sequence alignment; QDP: discovery confidence of the variant divided by the depth of coverage; SW: software.

Two separate and complementary pipelines (Materials and methods; Figure 1a), developed at Boston College (BC) and the BI, were used to identify SNPs in the sequence data. The main functional steps in both pipelines were as follows: (1) read mapping to align the sequence reads to the genome reference sequence; (2) alignment post-processing to remove duplicate sequence fragments and recalibrate base quality values; (3) variant calling to identify putative polymorphic sites; and (4) variant filtering to remove likely false positive calls.

#### Mapping

In both pipelines, the individual sequence reads were first mapped to the genome (using the entire human reference sequence, as opposed to just the targeted regions), with the MOSAIK [10] program (at BC), and a combination of the MAQ [11] and SSAHA2 [12] mapping programs (at BI) (Materials and methods).

#### Alignment post-processing

Mapped reads were filtered to remove duplicate reads resulting from clonal amplification of the same fragments during library construction and sequencing. If kept, such duplicate reads would interfere with variant detection. We also applied a base quality re-calibration procedure that resulted in a much better correspondence of the base quality values to actual base error rates (Figure S4 in Additional file 1), a property that is essential for accurate variant detection.

**Figure 2:**
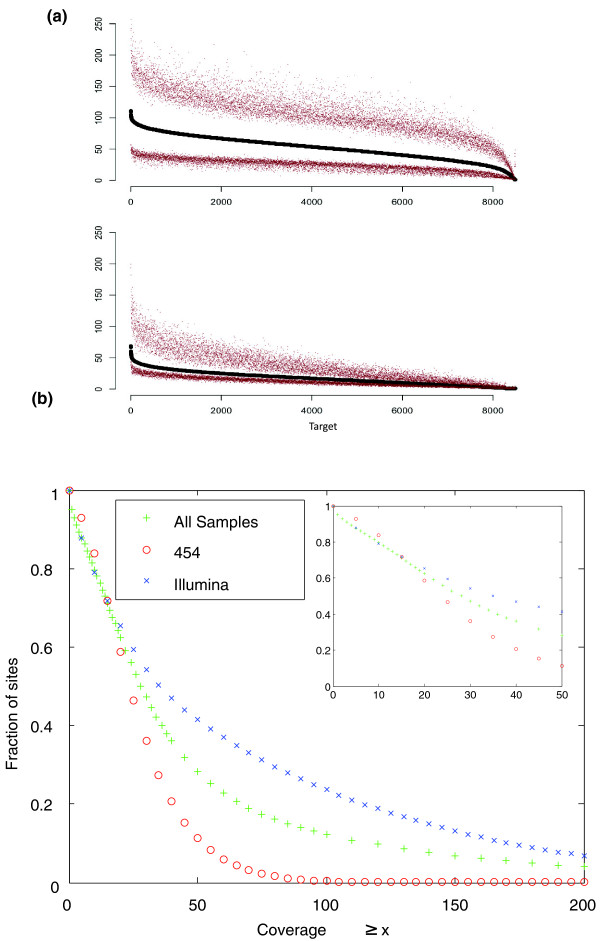
**Coverage distribution**. **(a) **Coverage across exon targets. Per-sample read depth of the 8,000 targets in all CEU and TSI samples. Targets were ordered by median per-sample read coverage (black). For each target, the upper and lower decile coverage value is also shown. Upper panel: samples sequenced with Illumina. Lower panel: samples sequenced with 454. **(b) **Cumulative distribution of base coverage at every target position in every sample. Depth of coverage is shown for all Exon Pilot capture targets, ordered according to decreasing coverage. Blue, samples sequenced by Illumina only; red, 454 only; green, all samples regardless of sequencing platform.

There was substantial heterogeneity in the depth of coverage of different regions that were targeted for capture (Figure 2a), reflecting different affinities for individual probes. Although the coverage variance was generally reproducible from experiment to experiment, additional variance could be attributed to individual samples, capture reagents, or sequencing platforms (Table 1). Despite this variance, >87% of the target sites in all samples have at least 5× read coverage, >80% at least 10×, and >62% at least 20× (Figure 2b).

#### Variant calling

The two pipelines differed in the variant calling procedures. Two different Bayesian algorithms (Unified Genotyper [13] at BI, GigaBayes at BC: see Materials and methods) were used to identify SNPs based on read alignments produced by the two different read mapping procedures. Another important difference between the BI and BC call sets was that the BI calls were made separately within each of the seven study populations, and the called sites merged *post hoc*, whereas the BC calls were made simultaneously in all 697 samples.

#### Variant filtering

Both raw SNP call sets were filtered using variant quality (representing the probability that the called variant is a true polymorphism as opposed to a false positive call). The BC set was only filtered on this variant quality and required a high-quality variant genotype call from at least one sample. The BI calls were additionally filtered to remove spurious calls that most likely stem from mapping artifacts (for example, calls that lie in the proximity of a homopolymer run, in low sequence coverage, or where the balance of reads for the alternative versus the reference allele was far from the expected proportions; see Materials and methods for more details). Results from the two pipelines, for each of the seven population-specific sample sets, are summarized in Table 2. The overlap between the two data sets (that is, sites called by both algorithms) represented highly confident calls, as characterized by a high ratio of transitions to transversions, and was designated as the Exon Pilot SNP release (Table 1). This set comprised 12,758 distinct genomic locations containing variants in one or more samples in the exon target regions, with 70% of these (8,885) representing previously unknown (that is, novel) sites. All data corresponding to the release, including sequence alignments and variant calls, are available through the 1000 Genomes Project ftp site [14].

**Table 2:** SNP variant calls in the seven Exon Pilot populations

	LWK	YRI	CHB	CHD	JPT	CEU	TSI	All 697
Unique to BC								
SNPs	580	716	925	831	983	613	448	1,384
%dbSNP	23.5	15.6	26.7	24.1	27.6	19.9	23.4	5.4
Ts/Tv	2.09	0.95	1.23	1.68	1.54	0.92	0.71	1.38
								
Both BC and BI								
SNPs	5,459	5,175	3,415	3,431	2,900	3,489	3,281	12,758
%dbSNP	50.1	53.8	52.6	50.3	57.9	65.9	65.6	30.36
Ts/Tv	3.67	3.56	3.74	3.64	3.67	3.47	3.53	3.82
								
Unique to BI								
SNPs	911	694	557	450	1,819	327	1,004	5,391
%dbSNP	9.8	10.2	5.8	6.4	1.7	15.9	4.8	3.13
Ts/Tv	1.56	1.48	1.37	1.33	0.74	1.32	0.85	1.05

Calls made by the Boston College pipeline only (unique to BC), calls made by the Broad Institute pipeline only (unique to BI), and calls made by both pipelines (both BC and BI) are reported. Ts/Tv, transition/transversion ratio.

### Specificity and sensitivity of the SNP calls

A series of validation experiments (see Materials and methods; Table S1 in Additional file 1), based on random subsets of the calls, demonstrated that the sequence-based identification of SNPs in the Exon Pilot SNP release was highly accurate. More than 91% of the experimental assays were successful (that is, provided conclusive positive or negative confirmation of the variant) and therefore could be used to assess validation rates. The overall variant validation rate (see Table S2 in Additional file 1 for raw outcomes; see Table S3 in Additional file 1 and Table 3 for rates) was estimated at 96.6% (98.8% for alternative allele count (AC) 2 to 5, and 93.8% for singletons (AC = 1) in the full set of 697 samples). The validation experiments also allowed us to estimate the accuracy of genotype calling in the samples, at sites called by both algorithms, as >99.8% (see Table S4 in Additional file 1 for raw outcomes; see Table S5 in Additional file 1 for rates). Reference allele homozygotes were the most accurate (99.9%), followed by heterozygote calls (97.0%), and then alternative allele homozygotes (92.3%) (Table S5 in Additional file 1). Although the main focus of our validation experiments was to estimate the accuracy of the Exon Pilot SNP release calls, a small number of sites only called by the BC or the BI pipeline were also assayed (Table S2 in Additional file 1). Although there were not enough sites to thoroughly understand all the error modes, these experiments suggest that the homopolymer and allele balance filters described above are effective in identifying false positive sites from the unfiltered call set.

**Table 3:** Validation outcomes and rates of the Exon Pilot SNP variant calls

	AC = any	AC = any	AC = 1	AC = 2 to 5	Totals
Samples	All 697	CEU + CHB + YRI	All 697	All 697	
Series	Series 1	Series 2	Series 3+4	Series 3 + 4	Series 1 to 4
Variant	92	122	166	164	544
Non-variants	3	3	11	2	19
Validation rate	96.8%	97.6%	93.8%	98.8%	96.6%

Outcomes and rates are reported for various alternate allele count (AC) ranges.

**Figure 3:**
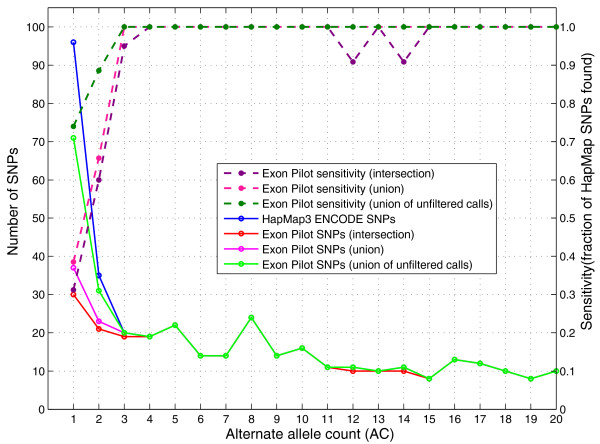
**Sensitivity measurement of Exon Pilot SNP calls**. Sensitivity was estimated by comparison to variants in HapMap, version 3.2, in regions overlapping the Exon Pilot exon targets. Circles connected with solid lines show the number of SNPs in such regions in HapMap, the Exon Pilot, and the Low Coverage Pilot project, as a function of alternative allele count. Dashed lines indicate the calculated sensitivity against the HapMap 3.2 variants. Sensitivity is shown for three sets of calls: the intersection between filtered call sets from BC and BI (most stringent); the union between the BC and BI filtered call sets; and the union between the BC and BI raw, unfiltered call sets (most permissive).

We performed *in silico *analyses (see Materials and methods) to estimate the sensitivity of our calls. In particular, a comparison with variants from the CEU samples that overlap those in HapMap3.2 indicated that our average variant detection sensitivity was 96.8%. A similar comparison with shared samples in the 1000 Genomes Trio Pilot data also showed a sensitivity >95% (see section 7, 'SNP quality metrics - sensitivity of SNP calls', in Additional file 1). When the sensitivity was examined as a function of alternative allele count within the CEU sample (Figure 3), most missed sites were singletons and doubletons. The sensitivity of the intersection call set was 31% for singletons and 60% for doubletons. For AC > 2, sensitivity was better than 95%. The strict requirement that variants had to be called by both pipelines weighted accuracy over sensitivity and was responsible for the majority of the missed sites. Using less strict criteria, there was evidence for 73% of singletons and 89% of doubletons in either the BC or the BI unfiltered dataset.

We investigated other, data-related determinants of singleton detection sensitivity, beyond the impact of the Project's decision to form the official Exon Pilot variant list as the intersection of the two independently derived call sets (see section 7.1, 'Sensitivity of singleton detection', in Additional file 1, and Figure S7 in Additional file 1). Singleton detection sensitivity improves significantly from low (1× to 9×) to medium (10× to 29×) read coverage (although there is no further improvement beyond 30× coverage). Importantly, approximately 9% (9 of 97) of HapMap3.2 singletons in the 84 samples shared with the Exon Pilot CEU sample panel had zero read coverage in our data. There was no significant difference in sensitivity between the Illumina and 454 reads, at comparable sequence coverage. Based on these observations, the main data-related reason for lower singleton sensitivity is lack of sufficient read coverage in the samples that have the singleton. Finally, our analysis (data not shown) revealed that, even at some of the sites with >100× read coverage in the sample with the putative HapMap3 singleton, there were no reads showing the alternative allele, and therefore it would not be possible to call the sites from the primary data. These cases represent either sites with allele-specific capture (that is, fragments with the alternative allele were not captured) or false positive sites in the HapMap3 study.

### Nucleotide diversity and allele frequency distributions

The high quality of the data enabled us to accurately estimate values of nucleotide diversity, a commonly used measure of genetic variability within populations, in the coding regions (using pair-wise heterozygosity as our metric (section 8, 'Heterozygosity estimates', in Additional file 1) within each of the seven populations (Table 1). These estimates were confirmed in the 1000 Genomes Low Coverage Pilot data in the Exon Pilot target regions (Table S9a in Additional file 1). Nucleotide diversity in the coding regions was 47.3 to 48.4% of the genome-averaged value for the corresponding population (Table S9b in Additional file 1). As expected, diversity was substantially higher in African than in European and Asian populations. It was, however, very similar for populations within the same continent (Table S9c in Additional file 1). Missense variation is substantially reduced (for example, compared to four-fold degenerate sites, where a single base substitution does not alter the amino acid) as a result of purifying selection. In turn, diversity at four-fold degenerate sites is comparable to average genomic diversity, consistent with very weak selection, if any. Diversity ratios across site types (for example, missense, four-fold degenerate) and datasets (for example, Exon Pilot, Low Coverage Pilot) are highly consistent between populations.

**Figure 4:**
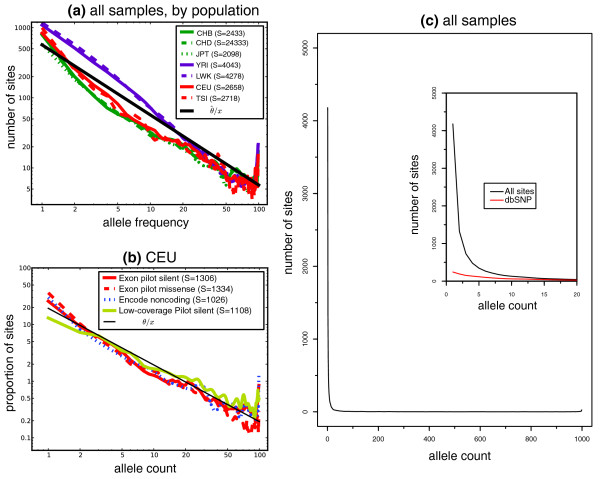
**Allele frequency properties of the Exon Pilot SNP variants**. **(a) **The allele frequency spectra (AFS) for each of the seven population panels sequenced in this study, projected to 100 chromosomes, using chimpanzee as a polarizing out-group. The expected AFS for a constant population undergoing neutral evolution, θ/x, corresponds to a straight line of slope -1 on this graph (shown here for the average value of the Watterson's θ nucleotide diversity parameter over the seven populations). Individuals with low coverage or high HapMap discordance (section 9, 'Allele sharing among populations', in Additional file 1) have not been used in this analysis. **(b) **Comparison of the site frequency spectra obtained from silent and missense sites in the Exon Pilot, as well as intergenic regions from the HapMap resequencing of ENCODE regions, within CEU population samples. The frequency spectra are normalized to 1, and S indicates the total number of segregating sites in each AFS. Individuals with low coverage or high HapMap discordance (section 9 in Additional file 1) have not been used in this analysis. **(c) **Allele frequency spectrum considering all 697 Exon Pilot samples. The inset shows the AFS at low alternative allele counts, and the fraction of known variant sites (defined as the fraction of SNPs from our study that were also present in dbSNP version 129).

We compared the allele frequency spectrum (AFS) in the sequenced coding regions among the Exon Pilot populations (Figure 4a). The high sensitivity assures us that the observed AFS are accurate for AC > 2 (or AF > approximately 1%). The AFS were very similar for populations from the same continent, except for the JPT population, where we observed a significantly lower fraction of rare alleles than in the two other Asian populations, consistent with reduced recent population growth in Japanese. Despite the large difference among continents at low AF, they converged at higher AF, reflecting the greater age of common variants, many of which pre-date the expansion of modern humans out of Africa. In all seven populations, there was a notable excess of rare variants compared to predictions for a constant-size, neutrally evolving population. This effect was enhanced at missense sites (Figure 4b), which were more highly represented at low alternative allele frequency than silent variants, as well as intergenic variants from the HapMap Encyclopedia of Coding Elements Project (ENCODE) re-sequencing study. The apparent excess of high frequency derived sites has often been observed in studies of human AFS, and may in part be due to ancestral misidentification [15].

### Rare and common variants according to functional categories

Recent reports [16] have also recognized an excess of rare, missense variants at frequencies in the range of 2 to 5%, and suggested that such variants arose recently enough to escape negative selection pressures [9]. The present study is the first to broadly ascertain the fraction of variants down to approximately 1% frequency across nearly 700 samples. Based on the observed AFS (Figure 4c), 73.7% of the variants in our collection are in the sub-1% category, and an overwhelming majority of them novel (Figure 4c, inset). The discovery of so many sites at low allele frequency provided a unique opportunity to compare functional properties of common and rare variants.

**Figure 5:**
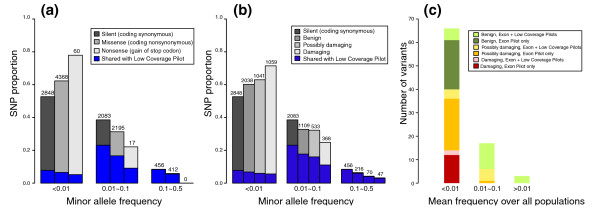
**The distribution of functionally characterized Exon Pilot SNPs according to minor allele frequency within all samples**. **(a) **Annotation according to amino acid change. The distribution of the Exon Pilot coding SNPs classified according to amino acid change introduced by the alternative allele (silent, missense, and nonsense) is shown, as a function of AF. Both missense and nonsense variants are enriched in the rare allele frequency bin compared to silent variants, with highly significant *P *<< 10^-16^. The differences remain significant after correcting for the differential error rates in different bins (*P *<< 10^-16 ^for missense, and *P *<< 10^-5 ^for nonsense). **(b) **Computational prediction of functional impact. The distribution of SNPs classified according to functional impact (benign, possibly damaging, and damaging) based on computational predictions by the SIFT and PolyPhen-2 programs, as a function of allele frequency. In case of disagreement, the more severe classification was used. Silent SNPs are also shown, as neutral internal control for each bin. The damaging variants are highly enriched in the rare bin compared to the silent variants with highly significant *P *<< 10^-16^. This remains significant after correcting for the differential error rates in different bins (*P *<< 10^-16^). (a-b) Allele frequency was binned as follows: low frequency, <0.01; intermediate frequency, 0.01 to 0.1; and common, >0.1. The fraction of SNPs also called in the 1000 Genomes Low Coverage Pilot is indicated by blue shading, in each category. **(c) **Functional impact among variants shared with HGMD. Functional predictions using SIFT and PolyPhen-2 for the variants shared between the Exon Pilot and HGMD-DM, as a function of the disease allele frequency bin (<0.01, 0.01 to 0.1, and >0.1). Color represents predicted damage (green, benign; orange, possibly damaging; red, damaging); open sections represent variants shared between the Exon Pilot and Low Coverage Pilot, while solid sections represent variants observed only in the Exon Pilot.

We used three approaches to classify the functional spectrum (see Materials and methods): (i) impact on the amino acid sequence (silent, missense, nonsense); (ii) functional prediction based on evolutionary conservation and effect on protein structure by computational methods (SIFT [17] and PolyPhen-2 [18]); and (iii) presence in a database of human disease mutations (Human Gene Mutation Database (HGMD)). All three indicators showed a substantial enrichment of functional variants in the low frequency category within our data (Figure 5). First, and as noted by other studies [19,20], we saw a highly significant difference (*P *<< 10^-16^) in the AFS of silent versus missense variants (Figure 5a) with a skew towards rare alleles in the latter, so that approximately 63% of missense variants were <1% in frequency whereas approximately 53% of silent variants fell into this category. The same patterns held for nonsense versus either silent or missense variants (*P *<< 10^-16^) where approximately 78% of nonsense variants were below AF = 1%. Second, we found that PolyPhen-2/SIFT damaging predictions (Figure 5b) were likewise enriched in the rare part of the spectrum (approximately 72% for damaging versus 63% for possibly damaging, and 61% benign). This observation goes an important step beyond the enrichment of amino acid changing variants because the PolyPhen-2/SIFT programs make specific predictions about whether or not such a variant is damaging to protein function. Error rate variation between different AFS bins was not a significant confounder for these conclusions: error rates were estimated at 6.2%, 3.2% and 3.4% for different AFS bins (Tables S3, S4 and S5 in Additional file 1) and highly significant differences were still found after correcting for this error rate variation (*P *<< 10^-16 ^for missense, and *P *< 10^-5 ^for nonsense SNPs). Third, 99 coding variants in our dataset were also present in HGMD, and therefore linked with a disease in the literature (although not necessarily causative). We tested these variants with SIFT and PolyPhen-2, and obtained predictions for 89 (Figure 5c). All 14 variants classified as damaging were below 1% frequency in our dataset, and found only in a heterozygous state. This observation strongly suggests that the majority of variants that are directly damaging to protein structure and therefore may result in deleterious phenotypic effects (that is, actual causative variants, as opposed to merely disease-linked markers) are likely to occur at low AF in the population. It is also noteworthy that only a very small fraction (<20% in each category, marked on all three panels of Figure 5) of the putatively damaging variants in the Exon Pilot dataset were detected with an alternative, low coverage whole genome sampling strategy employed in the Low Coverage Pilot in the 1000 Genome Project [19], which was designed to find common variants but not powered to systematically detect low frequency sites (also see Figure 4b). The higher performance in detecting rare damaging variants in the Exon Pilot compared to the Low Coverage Pilot underlines the utility of targeted exome sequencing for disease studies.

### The extent of between-population allele sharing in rare versus common variants

**Figure 6:**
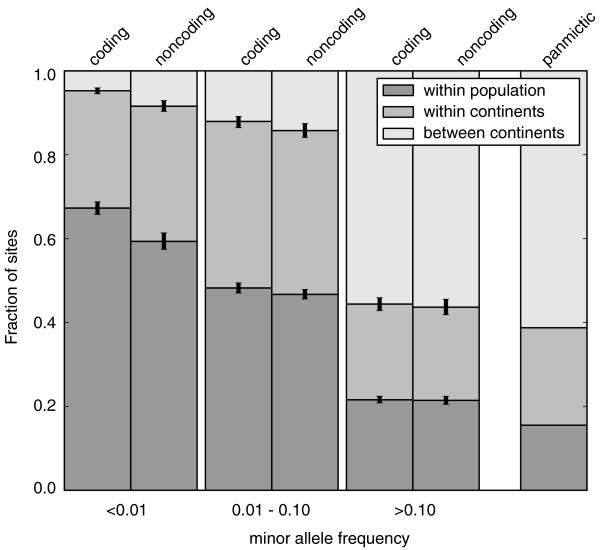
**Allele sharing among populations in the Exon Pilot versus ENCODE intergenic SNPs**. The probability that two minor alleles, sampled at random without replacement among all minor alleles, come from the same population, different populations on the same continent, or different continents, displayed according to minor allele frequency bin (<0.01, 0.01 to 0.1, and 0.1 to 0.5). For comparison, we also show the expected level of sharing in a panmictic population, which is independent of AF. The ENCODE and the Exon Pilot data have different sample sizes for each population panel, which could impact sharing probabilities. We therefore calculated the expected sharing based on subsets of equal size, corresponding to 90% of the smallest sample size for each population (section 9, 'Allele sharing among populations', in Additional file 1). To reduce possible biases due to reduced sensitivity in rare variants, only high-coverage sites were used, and individuals with overall low coverage or poor agreement with ENCODE genotypes were discarded. Error bars indicate the 95% confidence interval based on bootstrapping at individual variant sites.

We next examined the patterns of allele sharing (Materials and methods) among the Exon Pilot populations and between continents (Figure 6), and observed an expected reduction in the degree of allele sharing at low frequency. Comparison to intergenic variants from the HapMap3 ENCODE re-sequencing project [7] revealed that allele sharing at high and intermediate frequency was similar, but that at AF <1% it was substantially reduced in the coding regions, relative to intergenic regions (*P *< 10^-6^). This suggests that the low level of allele sharing of rare coding variants cannot be explained by allele frequency alone, and that such variants are likely to be younger than would be expected from neutral models, presumably because of negative selection acting at these sites.

### Short insertion/deletion variants in the Exon Pilot data

In addition to SNPs, the data also supported the identification of multiple, 1- to 30-bp insertions and deletions (INDELs; Materials and methods). The BCM and BI INDEL calling pipelines were applied (Figure 1b), and identified a total of 21 insertions and 75 deletions in the 1.43 Mb target regions (Tables S6 and S7 in Additional file 1). Comparisons with dbSNP and the other pilot projects showed high concordance rates. The overall experimental INDEL validation rate (Table S8 in Additional file 1) was 81.3%. Secondary visual inspection revealed that many of the events that did not validate were cases where multiple INDEL events were incorrectly merged, and the wrong coordinates were submitted for validation. This visual inspection confirmed all such alleles as true positives, substantially raising the effective validation rate. Coding INDEL variants change the amino acid sequence of the gene, and therefore these variants are very likely to impact protein function. Indeed, the majority of the events were non-frameshift variants (Figure S5 in Additional file 1) altering, but not terminating, the protein sequence. In agreement with our observations for SNPs, most INDELs were present at low population allele frequency (Figure S6 in Additional file 1).

## Conclusions

In addition to its goal of generating an extensive catalog of human population variations, the 1000 Genomes Project has served as an intensive technology development project in terms of both molecular methodologies and informatics methods for high-throughput data collection and data analysis. Although it is not a main focus of our manuscript, development and refinement of the DNA capture methods for this project have led to the current whole-exome capture reagents available for the community. The Exon Pilot project also led to the construction of informatics pipelines for effective analysis of targeted exon sequencing data, and these pipelines are now routinely used for whole-exome datasets. This study clearly lays out the informatics steps required to analyze such datasets and avoid the many pitfalls due to capture biases, coverage fluctuations, INDELs and alignment issues, population biases, and sequencing errors.

The extensive collection of SNPs in the 8,000 exons, detected with accurate and sensitive algorithms, allowed us to characterize fundamental variation properties in coding regions, and to compare them to overall genomic variation. The most important contribution of this study concerns the functional properties of rare variations, and their population specificity. We see a substantial depletion of putatively functional variants at intermediate and high AF, and a corresponding enrichment at low AF, which is expected as a result of negative selection, and has been noted recently [20,21]. However, our ability to study variants at 1% frequency revealed more direct signals, strongly suggesting that variants conferring direct changes on protein function will be present mostly at low population frequency. We were also able to note a significant reduction in the level of between-population allele sharing of rare coding variants, compared to intergenic variants, an effect that was not visible for variants above 1% in frequency. This effect is likely to reflect a combination of more recent origin and stronger negative selection for rare alleles in coding, compared to intergenic regions. Our complete dataset, including a list of SNP and INDEL variants with well characterized ascertainment properties is providing a useful substrate for more specialized analyses [22] to interpret functional and population aspects of low frequency coding variation.

## Materials and methods

### Data collection

#### Baylor College of Medicine

NimbleGen 385 K capture chips were designed to target the coding regions of the 1,000 genes. Target enrichment was performed following the Short Library Construction Protocol and NimbleGen Arrays User's Guide. Capture libraries were then sequenced on the 454 FLX/Titanium platform using standard vendor emPCR, enrichment and sequencing methods (GS FLX Titanium Sample Preparation Manual).

#### Broad Institute

Single-stranded RNA 'bait' was produced using the Agilent microarray-based method. Genomic DNA was sheared and ligated to Illumina sequencing adapters. This 'pond' of DNA was hybridized with an excess of bait in solution. The sequencing was done using the Illumina GA-II sequencers to produce either 36-bp fragment reads or 76-bp paired-end reads.

#### Sanger Institute

A custom Nimblegen 385-K array was used following the manufacturer's protocols (Roche/Nimblegen, Madison, Wisconsin, USA), with the modification that no pre-hybridization PCR was performed. Captured libraries were sequenced on the Illumina GA platform as paired-end 37-bp reads.

#### Washington University in St Louis

Whole genome shotgun libraries for Illumina sequencing were prepared according to the manufacturer's instructions. The pool of synthetic oligos was amplified by PCR and incorporated biotin-14-dCTP to produce a biotinylated capturing library. Each target library was hybridized with the biotinylated capturing library, isolated using streptavidin magnetic beads, and then amplified by PCR. The captured library fragments were reclaimed by denaturation and sequenced as fragment end reads on the Illumina GAIIx sequencer.

### Derivation of a consensus capture target list

A substantial amount of technological heterogeneity existed among different centers' production pipelines. The Exon Pilot initially selected 1,000 genes as targeted sequences. However, the capture target designs used in the four production centers were significantly different. To account for the heterogeneity introduced by different capture designs, we defined a set of consensus exon target sequences by intersecting the initial designs (the individual .bed files) with the exonic sequences based on the CCDS database to create the consensus exon target sequences (Figure S2 in Additional file 1), which form the basis of all the analyses described in this study. The consensus has approximately 1.43 Mb of exonic sequence, covering 86.1% of the coding regions in the initial 1,000 genes (the consensus target definition file is available through the 1000 Genomes Project technical release ftp directory [23].

### Data processing and SNP calling procedures

The SNP calls were a result of intersecting SNP calls from the BI using the GATK [13] and from BC using the MOSAIK [24] read mapper and the GigaBayes variant detection algorithm [25] (a new version of the PolyBayes SNP discovery program [26]). The BC call set was generated by calling all 697 individuals together, and per-population call sets were generated by a straightforward projection algorithm: a variant was called in a population if at least one individual in the population carried a non-reference allele (Figure 1a). The BI calls were made separately within each of the seven populations and a superset call set was generated as the union of all seven individual population call sets (Figure 1a). Variants were only called in the consensus target regions.

#### Boston College SNP calling pipeline

##### Read mapping

MOSAIK hash size was 15 with minimum mismatches of 4, 6, and 12 for 36-, 51-, and 76-/101-mer read lengths. MOSAIK parameters for Roche 454 reads were set to 15 with at least 70% of the read being aligned with a 5% mismatch rate.

##### Duplicate marking

MOSAIK Illumina alignments were duplicate-marked using the MarkDuplicates program from the Picard software suite [27]. MOSAIK Roche 454 alignments were duplicate-marked with BCMRemoveDuplicates program (M Bainbridge, personal communication).

##### Base quality value recalibration

MOSAIK Illumina alignments were re-calibrated using GATK [13] (with the CountCovariates and TableRecalibration commands). Roche 454 reads aligned with MOSAIK were not recalibrated.

##### Bayesian SNP calling

GigaBayes was used at BC for SNP calls. Briefly, it calculates genotype likelihoods, excluding reads with a mapping quality of <20 and nucleotides with a base quality <20. It then calculates genotypes using the previously calculated genotype likelihoods and a prior on variant frequency. Summing the probabilities of sample genotypes with at least one non-reference allele generates the posterior probability.

##### SNP filtering

Variant calls were filtered out if they did not meet the criteria of a PHRED scaled quality score of at least 40 with at least one individual with a non-reference genotype with a genotype quality score of at least 10.

#### Broad Institute SNP calling pipeline

The Broad Institute employed a five-step protocol consisting of alignment, PCR duplicate marking, base quality score recalibration, application of the SNP calling algorithm, and filtration of the results.

##### Alignment with MAQ/SSAHA2

Reads were aligned by the Sanger Institute using MAQ and SSAHA2 for Illumina and Roche 454 data, respectively. All aligned reads and metadata (sequencing center, sequencing technology, run identifier, lane identifier, library identifier, and so on) were written in BAM format.

##### Duplicate marking

We applied the Picard [27] MarkDuplicates algorithm. This algorithm locates reads from the same sequencing library with precisely the same starting position on the genome. When more than one read is found to have the same start position, all but one are flagged as duplicates in the BAM file and therefore ignored in downstream processing.

##### Base quality score recalibration

To correct for inaccuracies in the base quality scores, we developed and applied a base quality score recalibrator. Comparison of the estimated quality scores to the empirical quality scores allowed us to compute corrected quality scores, which were recorded in the BAM files.

##### SNP calling

We developed a multi-sample Bayesian SNP calling algorithm, now part of the GATK package [13]. This algorithm considers reads from the provided samples simultaneously, attempting to ascertain the likelihood of a site harboring an alternative allele with a frequency of at least 1/N, where N is the number of samples provided. Once the presence of a variant is established, the likelihood for each sample's genotype is determined by a greedy combinatorial search algorithm (approximately behaving like Expectation-Maximization).

SNP calls were generated per population. The specific parameters used were: minimum base quality, 10; minimum mapping quality, 10; minimum confidence threshold, 50.

##### SNP filtering

The SNP calling stage provided a list of any site in the target region that may plausibly be variant. These sites were then filtered to identify a set of true variants, discarding the ones deemed to be false-positives. To this end, we developed several heuristic filters by comparing the behavior of different covariates for known variants versus novel variants. Putative variants failing the following filters were ignored in downstream analysis: QD (discovery confidence of the variant/depth of coverage) ≥5; HRun (length of adjacent, allele-sharing homopolymer run) >3; AB (allele balance of variant, averaged over all heterozygous samples, polarized for the reference allele) ≥75%; SnpCluster (N or more variants found within M bases of each other) 3, 10.

#### Intersecting the Boston College and Broad Institute call sets

Next, we intersected the BC and BI SNP call sets within the target consensus regions (Figure 1a). This intersecting operation greatly improved the SNP call accuracy (Table 2), and the calls within the intersection were used in our official Exon Pilot release in March 2010. Table 2 presents the SNP calls of the seven population-specific call sets (that is, CEU, TSI, CHB, CHD, JPT, LWK, and YRI) that were generated by BC and BI pipelines independently. Across each of the seven populations, the intersection calls (BC ∩ BI) range from 50 to 79% of the total SNP calls made by BC and BI; more than 50% of the calls were in dbSNP (build 129), and show a high transition/transversion ratio (Ts/Tv) above 3.00. The large fraction of overlapping SNPs, with a high fraction of dbSNP entries and high Ts/Tv ratio, indicated high quality in the intersection call sets. These call sets were thus highly confident due to being generated from two independent pipelines with quite different and complementary algorithms. Several iterations of comparisons and tuning of the pipelines led to convergence of these call sets. In addition, the intersection call sets have yielded high validation rates (Table 3; Table S2 in Additional file 1).

The BC unique SNP call set (BC\BI) or BI unique SNP call set (BI\BC) accounted for the remaining 30 to 50% of the SNPs. About 20% of BC unique calls and 8% of BI unique calls were present in dbSNP build 129. Both unique call sets had a much lower Ts/Tv of 1.00, indicating relatively lower quality in the unique call sets (Table 2).

### SNP call set validations

We designed five series of validation experiments in order to examine the false positive and false negative rate, both globally in the officially released call sets, and in the SNP calls specific to the BC or BI call set, as well as in the rare and singleton SNPs and almost all the SNPs altering codons (Table S1 in Additional file 1). The validation experiments were carried out at the BCM Human Genome Sequencing Center (BCM-HGSC) and BI, using PCR-Sanger sequencing and Sequenom genotyping, respectively.

#### Series 1 - random sampling

We randomly chose 105 non-dbSNP sites in the intersection (that is, regardless of the frequency spectrum), and tested them by Sequenom at BI across the entire sample set.

#### Series 2 - population-specific discovery

Approximately 135 non-dbSNP sites were chosen regardless of the frequency spectrum from each of CEU, YRI + LWK, and CHB + CHD + JPT populations. They were selected to represent both the BC/BI intersection, BC-specific and BI-specific call sets. The sites were genotyped using Sequenom at BI across the samples in the populations where they were discovered.

#### Series 3 - low frequency sites and false positives

We tested 510 sites at low frequency (1 to 5 alleles/occurrences; approximately 300 in the intersection and approximately 200 in the BC-specific/BI-specific sets) using PCR and Sanger sequencing at the BCM-HGSC, in the particular samples where they were discovered. We allocated approximately 50% of the sites to singletons, and approximately 50% to sites with alternative allele count 2 to 5.

#### Series 4 - low frequency sites and false negatives

We chose 33 sites with alternative allele count 2 to 5 and 35 singletons from the intersection call set, and tested across all samples using Sequenom at BI.

#### Series 5 - comparative categories

We drew 227 sites at low frequency (singletons and SNPs with an alternative allele count of 2 to 5) from different functional annotation classes (such as missense, silent, promoter regions, and so on), and examined them using PCR-Sanger sequencing at the BCM-HGSC.

### SNP validation rate and genotype accuracy estimation

The overall validation rate in the official released data set (that is, the intersection) was very high at 96.8% (Table 3; Tables S3 and S4 in Additional file 1), meeting and exceeding the 1000 Genomes Project goal of >95% validation. The validation rates at the low-frequency categories were also high, greater than 93.0% for singletons and SNPs with alternative allele count 2 to 5 (series 3, 4 and 5 in Table S2 in Additional file 1). The exceedingly high validation percentages indicated that 1) the high coverage targeted resequencing methods were effective in accurately detecting SNPs at both common and rare allele frequencies; and 2) the intersection calls were highly accurate, and the vast majority of correctly called low frequency alleles were indeed at low frequency. Most of the non-validated sites (Table S2 in Additional file 1) were in the unique fractions of the BC and BI call sets.

The genotype call accuracies were calculated by comparing the called genotypes to the genotype measurements in the validation assays for all four series (series 1 to 4; Table S5 in Additional file 1). In total, 33,938 called genotypes were compared, and the vast majority of the genotypes agreed with the validation results: 32,532, 1,320 and 12 for Ref/Ref (Homozygote Reference), Ref/Alt (heterozygote) and Alt/Alt (Homozygote NonReference) classes, respectively. The accuracy rate for all called genotypes was as high as 99.8%, with 99.9% accuracy for Homozygote Reference (HomRef), 97.0% for heterozygote (Het), and 92.3% for Homozygote NonReference (HomNonRef). The overall false discovery rate of variant genotypes was <3% and the missed variant genotype rate was <1% as measured in series 1. The variant genotypes in low-frequency categories in series 3 were confirmed for 133 of 133 (100%) singleton sites, and 395 of 419 (94.3%) SNPs with alternative allele count 2 to 5. The accuracy compared to series 4 validated sites showed the false discovery rate for these categories was approximately 6.0% with a missed variant genotype rate of 0.1%.

### Nucleotide diversity estimation

Per-base heterozygosity estimates for the Exon Pilot were calculated at missense, two-fold, three-fold, and four-fold degenerate sites, and all base pairs in the autosomal targeted regions. We included only targeted base pairs with ≥10× coverage in at least 100 chromosomes based on the MOSAIK alignments. The same analysis was performed on the Low Coverage Pilot, but excluding base pairs that were masked in the Low Coverage callability files [28]. Base pairs were masked if >20% of Illumina reads had a mapping quality of 0 and/or read depth was greater than twice the average depth at HapMap3 sites. Also, a base pair had to be callable in all three Low Coverage populations in order to be included in our analysis. Per-base estimates of heterozygosity of ENCODE regions in HapMap3 were normalized by the nominal sequence length of 1 Mbp.

Degeneracy was calculated based on the hg18 reference sequence and the Gencode gene model annotations [23]. Note that some base pair positions may have been counted in multiple categories due to differing reading frames in alternative splice variants at a locus, but this number was less than 1% in each category and should have negligible effects on the resulting analyses.

### Spectrum analysis

In the Exon Pilot SNP data set, not all variant sites had the same number of genotypes in each of the seven populations studied. In order to make comparisons of spectra from different populations easier, the unfolded AF spectrum (using orthologous bases from the panTro2 assembly as the ancestral alleles) for each population was projected to a common sample size of 100 chromosomes using the software Dadi [29]. The projection is based off the hyper-geometric distribution, without correcting for ancestral misidentifications.

### Analysis of predicted impact on gene function

#### Functional prediction

SIFT and PolyPhen-2 were used to predict possible impacts of missense SNPs on the function of human proteins. Both programs utilize sequence and/or structure information in prediction. SIFT uses sequence homology to build a position-specified scoring matrix with Dirichlet priors, whereas PolyPhen-2 uses both phylogenetic and structural features combined with machine learning. In total, 3,708 and 5,990 missense SNPs in the Exon Pilot were evaluated by either SIFT or PolyPhen-2. We evaluated 3,176 missense SNPs by both SIFT and PolyPhen-2, which had a concordance rate in functional prediction of 55%.

#### Functional analyses of Exon Pilot variants found in the HGMD

The overlaps of the Exon Pilot SNP and INDEL sets with the HGMD Professional 2009.4 version missense/nonsense SNPs, small insertions, small deletions and small INDELs were identified based on their locations in the reference genome sequence (build 36). There were no overlapping insertions, deletions or INDELs; however, 99 overlapping SNPs within the HGMD-DM class were found, and these were used in subsequent analyses. Four led to premature stop codons and the remaining 95 to missense amino acid changes; the consequences of these for protein structure were predicted using SIFT and PolyPhen-2. The predicted consequences were combined into three classes: (1) Benign: 'benign' from PolyPhen-2 + 'tolerated' from SIFT, or one of these plus no prediction from the other program; (2) Possibly damaging: 'possibly damaging' from PolyPhen-2 plus 'damaging (low confidence)' from SIFT, or a conflict between the predictions; (3) Damaging: 'probably damaging' from PolyPhen-2 plus 'damaging' from SIFT, or one of these plus no prediction from the other program. AFs were determined in each population from the number of disease and non-disease allele calls, excluding individuals with missing data. These AFs were averaged across all populations.

### Analysis of allele sharing within and across populations

Allele sharing was measured as a function of alternative allele frequency using the following steps. Singletons, which cannot be shared, were removed from the catalog of 12,758 Exon Pilot exonic variants. The remaining 7,137 variants were further filtered using stringent coverage requirements (section 9, 'Allele sharing among populations', in Additional file 1) to ensure that coverage fluctuations between populations would not impact sampling. As a measure of sharing, we considered the likelihood that two minor alleles, when sampled at random without replacement among all minor alleles, belonged to the same population, to different populations from the same continent, or to different continents. In a panmictic population, every pair of sampled chromosomes is equally likely to be sampled, and the expected sharing depends only on the number of pairs of chromosomes in each sharing category - a combinatorial property of sample sizes, but independent of allele frequency.

We compared the Exon Pilot data with published data obtained by resequencing ten 100-kb ENCODE regions as part of the International HapMap 3 Consortium study. We extracted 3,618 HapMap SNPs based on a noncoding annotation. Since the HapMap and Exon Pilot data differ in their sample sizes, we calculated the expected amount of sharing for each dataset based on subsampling each population panel to 90% of the minimum population size between the two datasets, namely CEU:134, CHB:162, CHD:54, JPT:152, LWK:108, TSI:98, YRI:170. The probability of sharing was averaged over all sites, weighted by the probability that a site had two minor alleles in the down-sampled set. Confidence intervals were obtained by bootstrap over the different variant sites.

### INDEL detection and analysis

INDELs were called on the Exon Pilot data from both the Illumina and the Roche 454 platforms, and the results were merged to create the final call set (Figure 1b). Only INDELs inside the consensus target regions were included in the official release. The Illumina data were processed with two independent pipelines in a parallel fashion, by BCM-HGSC and BI (Figure 1b; Table S7 in Additional file 1). The Roche 454 INDELs were processed by BCM-HGSC. The results were combined by taking the union of the three call sets (Figure 1b; Table S7 in Additional file 1).

#### BCM-HGSC Illumina INDEL calling pipeline

##### Read mapping

The BCM-HGSC Illumina INDEL calling pipeline used the MOSAIK alignments created at BC as explained in the SNP calling methods.

##### Duplicate filtering

Duplicate reads were marked in the alignment using the Picard MarkDuplicates tool [27] as explained in the SNP calling methods.

##### Base quality recalibration

The base qualities reported by the instrument were recalibrated using GATK as explained in the SNP calling methods.

##### INDEL calling

INDELs were called using Atlas-Indel2 (Challis *et al*., submitted), which uses logistic regression models trained on validated exon capture data to identify true INDELs and remove false INDELs arising from sequencing or mapping errors.

##### INDEL filtering

INDEL calls were further filtered to require at least two variant reads in a sample. We additionally filtered out all singleton INDELs with a length of 1, in order to remove the high number of false positive INDELs in this category.

#### Broad Institute Illumina INDEL calling pipeline

##### Read mapping

The BI Illumina INDEL calling pipeline used the MAQ alignments created at Sanger as explained in the SNP calling methods.

##### Duplicate filtering

Duplicate reads were marked in the alignment using the Picard MarkDuplicates [27] as explained in the SNP calling methods.

##### Base quality recalibration

The base qualities reported by the instrument were recalibrated using GATK as explained in the SNP calling methods.

##### Multiple sequence alignment near putative INDELs

Reads in the alignment were realigned by GATK IndelRealigner around putative INDELs.

##### INDEL calling

INDELs were called using IndelGenotyperV2.

##### INDEL filtering

INDEL calls were further filtered based on local mismatch rate, nearby homopolymer runs, strand bias and other similar features.

#### BCM-HGSC Roche 454 INDEL calling pipeline (Figure 1b)

##### Read mapping

The Roche 454 INDEL data were aligned using BLAT-CrossMatch at the BCM-HGSC.

##### Duplicate filtering

Duplicate reads were removed from the alignment using the BCMRemoveDuplicates script.

##### INDEL calling

INDELs were called using the Atlas-Indel program at the BCM-HGSC.

##### INDEL filtering

Initial calls were further filtered by removing lower quality reads, singleton INDELs, 2-bp low frequency INDELs, and any INDELs that may have arisen due to flow-space errors.

#### Merging INDEL call sets (Figure 1b)

The intersection of the BCM-HGSC and BI Illumina INDEL call sets was taken as the consensus for the Illumina data. The union of the Illumina consensus set and the Roche 454 call set formed the final call set. When merging call sets any INDELs of the same type (insertion or deletion) within 5 bp of each other were considered equivalent and merged together.

In total, we detected 96 INDELs (21 insertions and 75 deletions) from the 697 individuals (Table S7 in Additional file 1). The call set had a dbSNP (build 129) concordance rate of 26%. On the Illumina platform, 9 insertions and 39 deletions were called by BCM-HGSC and 11 insertions and 37 deletions by BI. A total of 10 insertions and 24 deletions were called on the Roche 454 data. The Roche 454 INDEL set appeared to be enriched with 2-bp INDELs. This is likely due to flowspace errors on the sequencing platform, which may make 1- or 3-bp INDELs appear to be 2 bp long.

When combining call sets from BCM-HGSC and BI, and calculating concordance, INDELs within 5 bp of each other and of the same type (insertion or deletion) were considered equivalent. The INDEL call set for each population was combined by continent for the alternative allele count analysis, and all seven sets were combined into one set for the INDEL size analysis (Figures S5 and S6 in Additional file 1). When INDELs were found to be equivalent, they were combined to remove the duplication. When combined to the continental level, 51 INDELs were found in Africa, 46 in Asia, and 30 in Europe (Figure 1b; Table S7 in Additional file 1).

### INDEL validation

The Illumina union INDEL calls were assessed by two methods (Table S8a in Additional file 1). First, the 31 INDELs called by both centers were validated via Sequenom assays for the haplotypes resulting from the INDEL event. The assays were designed using the GATK, and dbSNP sites were masked to avoid bias due to nearby SNPs. The 13 INDELs exclusive to the JPT population and exclusive to a single center were validated via Sequenom assays following the same protocol. Second, the remaining INDEL sites unique to either center were validated by targeted resequencing using PCR and the Roche 454 platform.

Sequenom probe design resulted in probes for 31 sites in the overlapping call set, of which five failed quality control checks. The remaining 26 sites all validated as variants, though genotype concordance between sequencing calls and validation was very low (Table S8b in Additional file 1). Of the 13 probes designed to assess the unique coding INDEL calls in the JPT population, 10 passed quality control filters, and 6 validated as true variants.

Unique INDELs from the Illumina BCM and BI call sets underwent PCR-Roche 454 validation at BCM-HGSC (Table S8a in Additional file 1). Some additional low-confidence INDELs that were filtered out of the BCM call set were also included for software tuning purposes. Equivalent INDELs within any of these sets were merged. A total of 114 on-target sample-sites were submitted for validation; 94 INDELs had conclusive results. The BI unique call set had a confirmation rate of 78.6% and the BCM-HGSC call set had a confirmation rate of 80.0% (Table S8c in Additional file 1). In addition to these INDELs, 405 off-target non-coding sample sites underwent validation. Of these, 227 gave conclusive results, BI INDELs had a confirmation rate of 88.6% and BCM-HGSC had a confirmation rate of 59.6%. BCM's low confirmation rate was due to the exon-specific nature of the Atlas-Indel2 pipeline.

## Abbreviations

AC: allele count; AF: allele frequency; AFS: allele frequency spectrum; BC: Boston College; BCM: Baylor College of Medicine; BCM-HGSC: Baylor College of Medicine Human Genome Sequencing Center; BI: Broad Institute; bp: base pair; CEU: Utah residents with Northern and Western European ancestry from the CEPH collection; CHB: Han Chinese in Beijing, China; CHD: Chinese in Metropolitan Denver, Colorado; ENCODE: The Encyclopedia of Coding Elements Project; HGMD: Human Gene Mutation Database; INDEL: insertion/deletion polymorphism; JPT: Japanese in Tokyo, Japan; LWK: Luhya in Webuye, Kenya; PCR: polymerase chain reaction; SNP: single-nucleotide polymorphism; Ts/Tv: transition/transversion ratio; TSI: Tuscans in Italy; YRI: Yoruba in Ibadan, Nigeria.

## Competing interests

The authors declare that they have no competing interests.

## Authors' contributions

GTM is co-chair of the 1000 Genomes Exon Sequencing Pilot Subgroup, designed the analysis, and coordinated the preparation of the manuscript. FY carried out much of the analysis and coordinated the supplement. ARI, KG, CH, WFL, MB, TB, YC, DC, DK, and ChS performed the data processing and variant calling. SG performed the population genetic analyses. CTS, EVB, DNC, CH, AW, JY, YC, and YX performed the functional impact analyses. CDB, AGC, and MD oversaw population genetic analysis. RG initiated and oversaw the project. DA, SG, EM, AP, and MDP oversaw data production and the data producing center's contributions. XZB and LC provided data management support, overseen by PF, KC, BF, DM, and RS, and CaS performed and oversaw data production. GTM, FY, CTS, SG, and RG wrote the manuscript. All authors read and approved the final manuscript.

## Supplementary Material

Additional file 1 **Supplemental information**. Additional methodological details, figures, tables and citations [30]. Click here for file

Additional file 2 **1000 Genomes Project members**. List of the member of the pilot phase of the 1000 Genomes Project. Click here for file
