# Supplementary material for: The functional spectrum of low-frequency coding variation
Source: Genome Biol. 2011 Sep 14;12(9):R84. doi: 10.1186/gb-2011-12-9-r84 (PMC3308047; doi:10.1186/gb-2011-12-9-r84)
Supplement: Additional file 1 — Supplemental information. Additional methodological details, figures, tables and citations [30]. [file gb-2011-12-9-r84-S1.DOC]

**SUPPLEMENTARY MATERIALS**

**Table of Contents and Display Items**

1. Introduction

Figure S1. Read depth of coverage comparison between the Exon Pilot and the Low-coverage Pilot.

2. Consensus exon target definition

Figure S2. The process to define the 1.43 Mb consensus target regions for data.

3. Data quality control (QC)

3.1 First iteration of data QC

3.2 Second iteration of data QC

Figure S3 Sample quality control.

4. Base quality recalibration

Figure S4. Base quality recalibration plots.

5. SNP validation

Table S1. Exon Pilot SNP validation design and success rate.

Table S2. SNP validation outcomes.

Table S3. SNP calling accuracy for the BC and BI Intersection calls.

Table S4. Genotype call accuracy in counts for the BC and BI Intersection calls.

Table S5. Genotype call accuracy rates for the BC and BI Intersection calls.

6. Insertion-deletion (INDEL) calling summary and analysis

6.1 INDEL calling from BCM and BI pipelines

Table S6. INDEL calls by BCM-HGSC and BI.

Table S7. Summary of the Exon Pilot INDEL call set.

6.2 INDEL analysis results

Figure S5. The INDEL size distribution of the call set.

Figure S6. INDEL variant allele count distribution by continent, frameshift versus non-frameshift.

6.2 Experimental INDEL validations

Table S8 INDEL validation design and results.

7. SNP quality metrics – sensitivity of SNP calls

7.1. Sensitivity of singleton detection

Figure S7. Sensitivity of the Exon Pilot SNP calls as a function of allele frequency.

7.2. Per-sample sensitivity estimates

8. Heterozygosity estimates of Exon and Low Coverage Pilots

Table S9 Per base pair estimates of heterozygosity

9. Allele sharing among populations

**1. Introduction**

The 1000 Genomes Project was designed to drive second-generation high-throughput sequencing methods, the development of effective data processing and variant calling methods, and to produce a catalog of normal human variation down to approximately 1% allele frequency genome-wide, and even more comprehensive discovery (down to frequencies of 0.1 – 0.5%) in functional gene regions. Three pilot projects, representing alternative coverage strategies were included: low-pass sequencing of ~200 samples (the Low Coverage Pilot), deep sequencing of two mother-father-child trios (the Trio Pilot), and high-coverage targeted exon (the Exon Pilot) sequencing of ~700 samples. The Low Coverage Pilot has on average ~2-4 X read-depth coverage, whereas the Exon Pilot can have up to 100 X coverage in the targeted exonic sequences (**Figure S1**). This design was aimed to enable genome-wide polymorphism discovery in hundreds of samples, exhaustive discovery in a small number of deeply sequenced samples, and the detection of low-frequency variants in exons.

The 1000 Genomes Exon Pilot Project was designed to evaluate high-coverage sampling in a deep, multi-population sample set. The focus of our study is the Exon Pilot Project data, representing high-coverage target-capture based exonic sequencing of nearly 700 samples (**Table 1**). It allows the interrogation of common and rare variants in the targeted regions in a very deep sample cohort. The data therefore would provide information about the frequency distribution of rare functional variants. The Exon Pilot data set was collected collaboratively from four different data production centers - Baylor College of Medicine-Human Genome Sequencing Center, Broad Institute, Sanger Institute and Washington University at St. Louis – using different targeted capture and the second generation sequencing platforms.


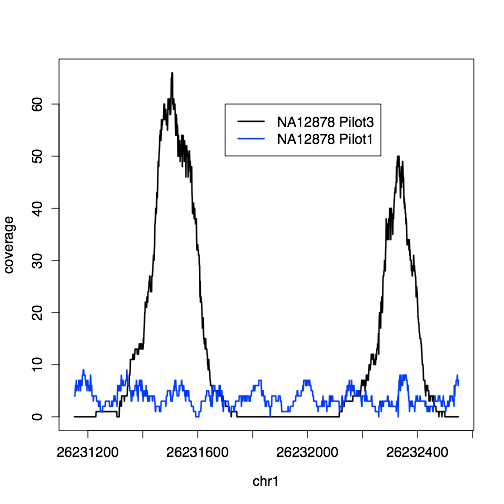


**Figure S1. Read depth of coverage comparison between the Exon Pilot (marked as Pilot3) and the Low-coverage Pilot (marked as Pilot1).**

**2. Consensus exon target definition**

The genomic coordinates of the target regions shared across the four data generating centers were derived according to the procedure described in the main text (**Methods**), and illustrated in **Figure S2** below.

**Figure S2. The process to define the 1.43 Mb consensus target regions for data.**

**3. Data quality control (QC)**

3.1 First iteration of data QC

The Exon Pilot working group monitored data quality issues during the course of the project. Based on the initial raw SNP call sets produced by each institute, an array of methods have been applied to the initial raw data set for data quality control at different centers. The process resulted in re-naming 19 samples that were identified as potential “sample swaps”, and withdrawing 28 samples that were identified as low quality possibly due to issues such as DNA cross-contamination.

*QC method 1.* For each comparison between one .fastq file and one candidate HapMap individual, we calculate an "excess mismatch rate" as: excess rate = (informative rate - background rate). The informative mismatch rate is the fraction of mismatches to the HapMap genotype data among all base calls mapped to sites where this candidate individual is homozygous, according to HapMap genotype data. The background mismatch rate is the fraction of mismatches to the reference sequence among all base calls mapped to sites that are not in dbSNP version 129 nor genotyped in HapMap.

*QC method 2*. This approach focuses on the variant alleles identified from the Exon Pilot, and compares them against the genotype data obtained from the HapMap Project. The BCM-HGSC has applied this method to the Roche 454 data sets. We firstly carried out a one-to-one concordance analysis for the 346 samples that also had HapMap genotype data, and used the concordance rate = 90% as the cutoff to narrow down the list of samples that might potentially have been swapped. We also ran another “all-against-all” for the remaining Exon Pilot samples that have no prior HapMap data. None of these have a high enough concordance rate to suggest a swap between these two groups of samples.

*QC method 3.* Broad Institute has applied this “all-against-all concordance method” to compute a score for each of the Illumina lanes against HapMap. If the HapMap sample with the best concordance (number of correct calls at HapMap sites / number of HapMap sites) is high (> 90%) and sufficiently better than the HapMap sample with the next-best concordance (> 15%), we take that first HapMap sample to be the true identity of the lane. If multiple HapMap samples have high concordance, we take that to be a lane failure.

3.2 Second iteration of data QC

After the variant data release in March 2010, another round of quality control was implemented to further identify any plausible sample-sample swap or contamination. In this, we compared the Exon Pilot SNP genotypes for each individual with the HapMap 3 genotypes and the Low-Coverage Pilot SNP calls. We computed the non-reference genotype mismatch percentage as our sample specific quality metric as a function of number of compared genotypes for each of the 697 samples. For example, **Figure S3** shows the variant genotype mismatch rate as a function of depth at the called genotypes in the JPT flagged sample NA18961. Regardless of the different SNP calling pipelines used at Boston College and Broad Institute, the genotype mismatch rates are always greater than 20% error rate when varying genotype depth (i.e., the numbers above the markers represent the minimum genotype depth thresholds). The read depth of coverage did not appear to be an issue, because most of these 17 flagged samples have >40 read depth of coverage per locus.

We applied a cutoff of 20% mismatch rate, and identified 17 samples (out of 697 samples in the Exon Pilot) with the mismatch rate greater than 20%. Specifically, the 17 samples are: NA12829 from CEU; NA18635, NA18642 and NA18749 from CHB; NA17966 and NA18670 from CHD; NA18948, NA18961, NA18964, NA19054, NA19065 and NA19068 from JPT; and NA18504, NA18516, NA18522, NA18870 and NA18871 from YRI.

We examined the effect of the 17 samples identified on the release SNP call set, and this was marginal. By removing the samples, the number of SNPs in each population was reduced by a marginal number, ranging from 30 to 50 SNPs. For most populations, most of these removed SNPs were known. However in YRI, most of the removed SNPs were novel and rare – possibly due to the ascertainment bias in dbSNP129 database. With respect to the frequency spectrum, the differences are almost exclusively confined to the low-frequency realm and insignificant.


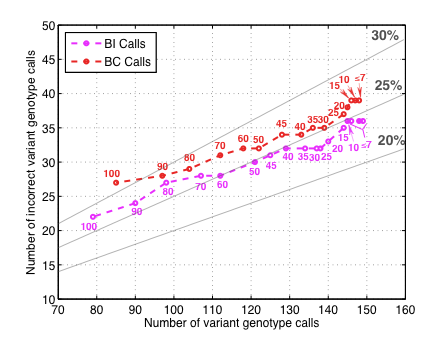


**Figure S3. Sample quality control.**

**4. Base quality recalibration.**

Base quality recalibration was performed according to the procedure described in the text, and illustrated in **Figure S4**.

**Figure S4. Base quality recalibration plots**. The correspondence between assigned and measured, phred-scaled base quality values are shown before (left) and after (right) recalibration.

**5. SNP validation**

**The details of the experimental validation methods are provided in the main text (Methods). The details of the validation results are shown in a series of tables below: Validation design and success rates (Table S1), validation outcomes (Table S2), SNP calling accuracy estimates (Table S3). The raw counts for the genotype calls are given in Table S4, and the derived accuracy estimates in Table S5.**

| **Series** | **Description** | **Populations** | **Samples** | **Designed Sites** | **Passed QC** | **Mono-morphic** | **Polymorphic** |
| --- | --- | --- | --- | --- | --- | --- | --- |
| Series 1 | Random sampling | All 697 | 694/697 | 105 | 95 (90%) | 3 | 92 (97%) |
| Series 2a | Population-specific discovery | CEU | 90/90 | 138 | 124 (90%) | 38 | 86 (69%) |
| Series 2b |  | CHB+CHD+JPT | 284/321 | 132 | 120 (91%) | 32 | 88 (73%) |
| Series 2c |  | LWK+YRI | 188/220 | 136 | 125 (92%) | 34 | 91 (73%) |
| Series 3 | Low freq. and false positives | All 697 | 605 | 510 | 468 (92%) | 83 | 385 (82%) |
| Series 4a | Low freq. and false negatives | All 697 | 694/697 | 33 | 30 (91%) | 0 | 30 (100%) |
| Series 4b | Singletons | All 697 | 694/697 | 35 | 34 (97%) | 1 | 33 (97%) |
| Series 5 | Comparative categories | All 697 | 605/697 | 227 | 207 (91%) | 5 | 202 (98%) |
| Total |  |  |  | 1316 | 1203 (91%) | 196 | 1007 (84%) |

**Table S1. Exon Pilot SNP validation design and success rate.**

| **Series** | **Samples** | **Category** | **Call Set** | **Variant** | **Non-Variant** | **Variant Rate** |
| --- | --- | --- | --- | --- | --- | --- |
| Series 1 | All 697 | AC = any | **BC ∩ BI** | 92 | 3 | 96.8% |
| Series 2 | CEU+CHB+YRI | AC=any | **BC \ BI** | 39 | 81 | 32.5% |
|  |  |  | **BC ∩ BI** | 122 | 3 | 97.6% |
|  |  |  | **BI \ BC** | 104 | 21 | 83.2% |
| Series 3 | All 697 | AC=1 | **BC \ BI** | 32 | 12 | 72.7% |
|  |  |  | **BC ∩ BI** | 133 | 10 | 93.0% |
|  |  |  | **BI \ BC** | 32 | 15 | 68.1% |
|  |  | AC =2-5 | **BC \ BI** | 19 | 31 | 38.0% |
|  |  |  | **BC ∩ BI** | 132 | 2 | 98.5% |
|  |  |  | **BI \ BC** | 37 | 13 | 74.0% |
| Series 4 | All 697 | AC=1 | **BC ∩ BI** | 33 | 1 | 97.1% |
|  |  | AC =2-5 | **BC ∩ BI** | 30 | 0 | 100% |
| Series 5 | All 697 | AC=1 | **BC ∩ BI** | 90 | 4 | 95.7% |
|  |  | R/R | **BC ∩ BI** | 90 | 0 | 100% |
|  |  | AC=2-5 | **BC ∩ BI** | 93 | 1 | 98.9% |

**Table S2. SNP validation outcomes.** The “*AC”* in the “*Category*” column means allele count.

|  | **AC = any** | **AC=any** | **AC=1** | **AC=2-5** | **Totals** |
| --- | --- | --- | --- | --- | --- |
| Samples | All 697 | CEU+CHB+YRI | All 697 | All 697 |  |
| Series | Series 1 | Series 2 | Series 3+4 | Series 3+4 | Series1-4 |
| Variant | 92 | 122 | 166 | 164 | 544 |
| Non-Variants | 3 | 3 | 11 | 2 | 19 |
| Validation rate | 96.8% | 97.6% | 93.8% | 98.8% | **96.6%** |

**Table S3. SNP calling accuracy for the BC and BI intersection calls.**

| **Validation series** | **Samples** | **Category** | **#called genotypes** | **Results** | | | | |
| --- | --- | --- | --- | --- | --- | --- | --- | --- |
| Series  1-4 | All 697 | AC=any | 33,938 |  | | **Validation** | | |
|  |  |  |  |  |  | **Ref/Ref** | **Ref/Alt** | **Alt/Alt** |
|  |  |  |  | **Call** | **Ref/Ref** | **32,532** | **31** | **1** |
|  |  |  |  |  | **Ref/Alt** | **39** | **1,320** | **2** |
|  |  |  |  |  | **Alt/Alt** | **1** | **0** | **12** |

**Table S4. Genotype call counts for the BC and BI Intersection calls.** The numbers in red represent numbers of called variant genotypes that failed validation; the numbers in green represent the validated variant genotypes; the numbers in blue represent the numbers of called non-variant genotypes where the validations indicated otherwise; and the number in black represents the validated non-variant genotypes.

| **Series** | **Samples** | **Category** | **FDR** | **MVGR** | **Genotype Accuracy** | | | |
| --- | --- | --- | --- | --- | --- | --- | --- | --- |
|  |  |  |  |  | **HomRef** | **Het** | **HomNonRef** | **All Genotypes** |
| Series 1 | All 697 | AC=Any | 2.8% | 0.1% | 99.9% | 96.9% | 90.9% | 99.8% |
| Series 2 | CEU | AC=Any | 0% | 0.03% | 99.9% | 100% | 100% | 99.9% |
|  | CHB | AC=Any | 1.5% | 0.1% | 99.9% | 98.5% | -- | 99.9% |
|  | YRI | AC=Any | 0.9% | 0.2% | 99.8% | 99.1% | 100% | 99.8% |
| Series 3 | All 697 | AC = 1 | 0% | -- | -- | 100 | -- | 100% |
|  |  | AC=2-5 | 5.7% | -- | -- | 94.3% | -- | 94.3% |
| Series 4 | All 697 | AC = 1 | 6.0% | 0.1% | 99.9% | 93.9% | -- | 99.8% |
|  |  | AC=2-5 | 0% | 0.01% | 99.9% | 100% | -- | 99.9% |
| **Series 1-4** | **All 697** | **AC=Any** | **2.9%** | **0.1%** | **99.9%** | **97.0%** | **92.3%** | **99.8%** |

**Table S5. Genotype call accuracy rates for the BC and BI Intersection calls.** The FDR is equal to number of called variant genotypes that fail validation, divided by the total number of called variant genotypes from the SNP caller. MVGR is equal to the number of called non-variant genotypes that fail validation, divided by the total number of called non-variant genotypes from the SNP caller. MVGR measures how many called non-variant genotypes are actually the variant genotype.

**6. Insertion-deletion (INDEL) calling summary and analysis**

6.1 INDEL calling from the BCM and BI pipelines

The raw INDEL calling results are shown in **Table S6**, and the released data set in **Table S7**.

|  | **YRI** | **CHB** | **CHD** | **JPT** | **CEU** | **TSI** | **LWK** | **Total** |
| --- | --- | --- | --- | --- | --- | --- | --- | --- |
| **Illumina BCM-HGSC insertions** | 7 | 4 | 2 | 6 | 4 | 6 | NA | 9 |
| **Illumina BCM-HGSC deletions** | 21 | 11 | 11 | 15 | 10 | 9 | NA | 39 |
| **Illumina BI insertions** | 4 | 5 | 3 | 5 | 5 | 4 | NA | 11 |
| **Illumina BI deletions** | 15 | 11 | 7 | 6 | 11 | 12 | NA | 37 |
| **Roche 454 BCM-HGSC insertions** | 4 | 6 | 5 | 3 | 2 | NA | 8 | 10 |
| **Roche 454 BCM-HGSC deletions** | 6 | 6 | 6 | 3 | 2 | NA | 16 | 24 |

**Table S6. INDEL calls by BCM-HGSC and BI.** INDELs were called on the Illumina Platform by BCM-HGSC and BI, and on the Roche 454 platform by BCM-HGSC. The union of these three sets created the final call set. The total column gives the total INDEL count, after combining all population call sets and merging equivalent INDELs.

**Africa**

**Asia**

**Europe**

**Indel Type**

**LWK**

**(dbSNP,**

**LowCov**

**,**

**Trio)**

**YRI**

**(dbSNP,**

**LowCov**

**,**

**Trio)**

**CHB**

**(dbSNP,**

**LowCov**

**,**

**Trio)**

**CHD**

**(dbSNP,**

**LowCov**

**,**

**Trio)**

**JPT**

**(dbSNP,**

**LowCov**

**,**

**Trio)**

**CEU**

**(dbSNP,**

**LowCov**

**,**

**Trio)**

**TSI**

**(dbSNP,**

**LowCov**

**,**

**Trio)**

**Total**

**Insertions**

8(4, NA, NA)

10(6,5,NA)

8(6,NA,NA)

8(5,5,NA)

7(5,6,4)

6(4,NA,NA)

21

**Deletions**

16(4, NA, NA)

29(12,17,12)

20(8,9,NA)

18(7,NA,NA)

17(9,10,NA)

16(8,11,5)

16(7,NA,NA)

75

**Africa**

**Asia**

**Europe**

**Indel Type**

**LWK**

**(dbSNP,**

**LowCov**

**,**

**Trio)**

**YRI**

**(dbSNP,**

**LowCov**

**,**

**Trio)**

**CHB**

**(dbSNP,**

**LowCov**

**,**

**Trio)**

**CHD**

**(dbSNP,**

**LowCov**

**,**

**Trio)**

**JPT**

**(dbSNP,**

**LowCov**

**,**

**Trio)**

**CEU**

**(dbSNP,**

**LowCov**

**,**

**Trio)**

**TSI**

**(dbSNP,**

**LowCov**

**,**

**Trio)**

**Total**

**Insertions**

8(4, NA, NA)

9(5,6,4)

**Deletions**

16(4, NA, NA)

**Table S7. Summary of the Exon Pilot INDEL call set.** Counts of insertions and deletions found in each population and the total number (merged) of INDELs across all populations. The call set from each population was compared with dbSNP (build 129) and the low coverage and trio pilots where applicable. The overlap from this comparison is shown in parenthesis.

6.2 INDEL analysis results

Due to the short read length of the sequencing platforms, only relatively small INDELs (1-12 base pairs) could be detected (**Figure S5**). Most of the 6-12 base pair INDELs called came from the Roche 454 platform, as it has a significantly longer read length than the Illumina data. The 12 base-pair insertion is an allele of one of the 6 base-pair insertions located on chromosome 14 at coordinate 22618637, found on the Roche 454 platform. In all pipelines there appears to be a bias towards deletions and against insertions. This bias is apparent in the mapping stage, so it is not an artifact of the INDEL calling pipelines. We reason that the bias against insertions is an inherent limitation of the capture-short read sequencing technologies and alignment procedures used in the exon pilot project.

Only 39.6% of the called INDELs cause frameshifts, showing a strong tendency towards being non-frameshift, as expected in exonic regions presumably because of the purifying selection on frameshift INDELs. Additionally, we suspect the currently detected frameshift INDELs may be overrepresented due to off-by-one flowspace errors on the Roche 454 platform, as it was highly enriched with 2 base pair INDELs.

Frameshift INDELs appear to have a lower variant allele count than non-frameshift INDELs (**Figure S6**). This difference however is not shown to be significant by a student’s t-test except in Europe, with p-values of 0.11 for Africa, 0.82 for Asia, and 0.03 for Europe. This lack of significance is likely due to the low INDEL count. These results are consistent with that frameshift INDELs in exonic DNA were under purifying selection pressure and therefore segregating at lower population frequency.

Comparing our results to other previous studies highlights the depletion of INDELs, and especially frameshift INDELs, within exonic regions. Across all populations, our INDEL call set has 1 INDEL per 14.3 kb, as compared to 1 INDEL per 7.2 kb (1 per 6.3 kb within genes including non-coding sequences) in Mills et al. . Our study confirms Mills et al.'s findings that frameshift INDELs are much less frequent than non-frameshift INDELs in coding DNA: in Mills et al.'s study, 38.9% of INDELs in coding DNA are frameshift INDELs, and in our study 39.6% of the INDELs are frameshift INDELs. These rates were much lower than the frameshift rates found in the Low Coverage Pilot and Trio Pilot, where the frameshift rates were 57.2% and 60.0% respectively .

**Figure S5. The INDEL size distribution of the call set.** Duplicate INDELs were merged across populations. As expected in exonic regions, frameshift INDELs are highly depleted. Due to short read length, only small INDELs are identified.

**Figure S6. INDEL variant allele count distribution by continent, frameshift versus non-frameshift.** The population INDEL callsets were merged by continent, the variant allele count for each INDEL was calculated and frameshift and non-frameshift INDELs were separated. "n" is the number of individuals in each analysis panel. A student's t-test was performed to measure the difference between frameshift and non-frameshift variant allele count. The resulting p-values are shown.

6.2 Experimental INDEL validations

The validation results for the Exon Pilot INDEL calls are reported in **Table S8** below.

a.

**Description**

**Method**

**Populations**

**Samples**

**Designed Sites**

**Regions**

Baylor/Broad overlap

Sequenom

All, Illumina data

only

336

31

All

Baylor/Broad unique

(selection)

Sequenom

JPT

89

13

Coding Only

Baylor/Broad unique

(all sites)

PCR

-

Roche

454

All, Illumina data

only

176

340

All

Baylor 1bp singletons

(outside call set)

PCR

-

Roche

454

All, Illumina data

only

79

100

All

Baylor low

confidence (outside

call set)

PCR

-

Roche

454

CEU and YRI

28

30

Coding Only

b.


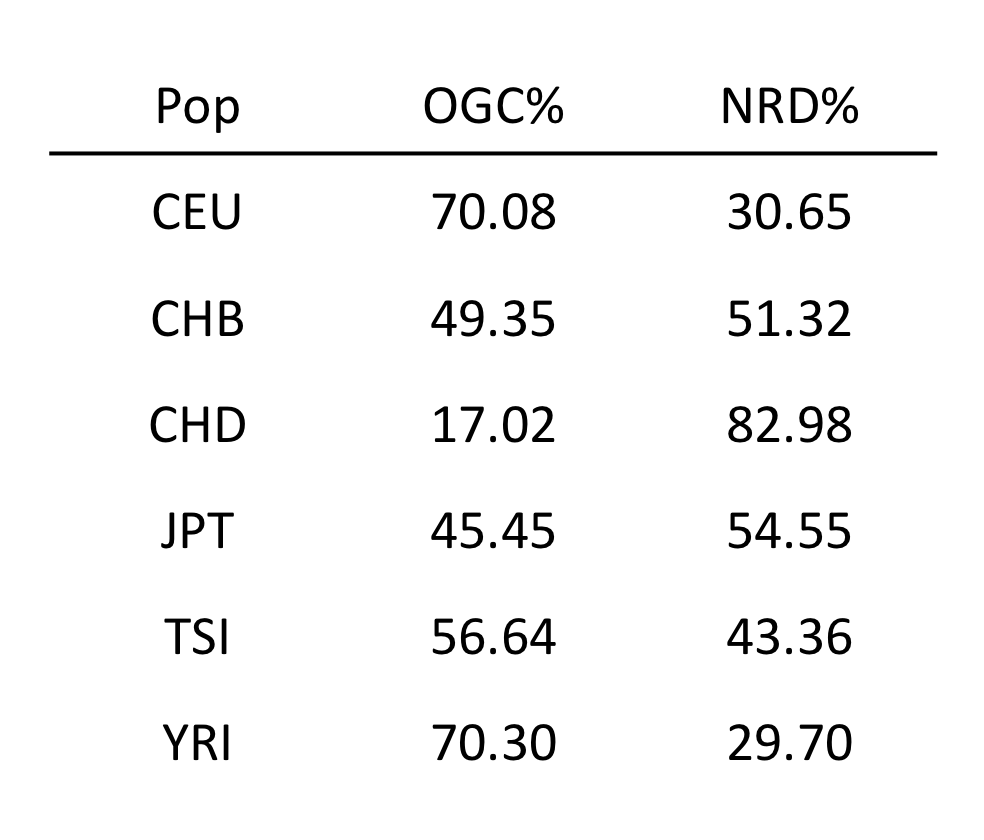


c.

| **Category** | **Confirmed** | **False Positive** | **Ambiguous/Failed** | **Confirmation Rate** |
| --- | --- | --- | --- | --- |
| BCM-HGSC | 28 | 7 | 5 | 80.0% |
| Broad Institute | 22 | 6 | 3 | 78.6% |
| BCM Low Conf. | 11 | 12 | 9 | 47.8% |

**Table S8. INDEL validation design and results.** (a) Summary of INDEL validations performed on the Illumina call sets. (b) Overall genotype concordance (OGC) and the non-reference discrepancy (NRD) rates in our INDEL genotypes based on the Sequenom validation results. (c) PCR-Roche 454 on-target (coding) validation results. INDELs are listed as either confirmed by the validation, proved to be a false positive by the validation, or ambiguous/failed if they failed in some step of the validation process. Equivalent INDELs are merged. Baylor 1bp singletons are included in the Baylor low confidence row.

**7. SNP quality metrics – sensitivity of SNP calls**

7.1 Sensitivity of singleton detection

We investigated the determinants of singleton detection sensitivity, beyond the impact of forming the intersection between the Boston College and the Broad Institute variant call sets (which results in 31% overall singleton sensitivity, as compared to 73%, when the most permissive call set, the union of BC and BI unfiltered calls are used, as discussed in the main text, see **Figure 3**). We used the 97 singleton sites in HapMap3.2 in the 84 samples shared with the 1000 Genomes Project Exon Pilot, and classified these sites according to read coverage, separately for Illumina and 454 reads, as well as in aggregate. We then calculated sensitivity according to the most permissive call set, the union between the BC and BI unfiltered calls (**Figure S7**). This analysis allowed us to investigate the impact of sequencing technology and read depth on singleton detection sensitivity. This analysis reveals that detection sensitivity improves as a function of read depth, from low (1-9X) coverage to intermediate (10-29X) coverage, but that there is no significant further improvement at high (>30X) coverage. Based on our data, we see no significant difference in sensitivity between the Illumina and the 454 data.


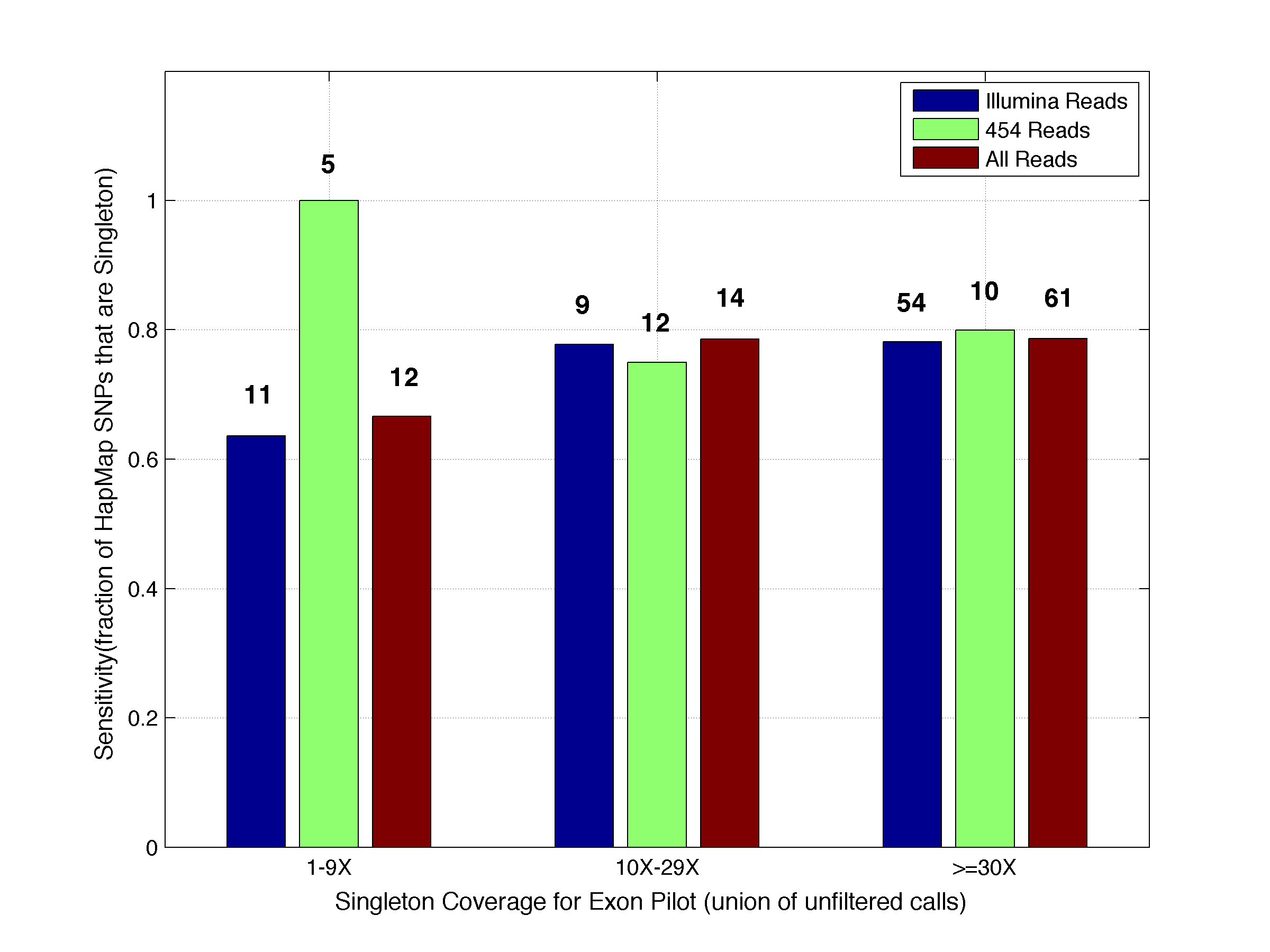


**Figure S7. Sensitivity of singleton detection as a function of sequencing technology and sequence coverage (read depth).** The underlying data represents the 97 singleton sites in HM3E, within 84 CEU samples shared between HM3E and the 1000 Genomes Project Exon Pilot. The singleton sites were binned according to read coverage in the Exon Pilot. The analysis was carried out considering coverage by Illumina reads only (blue), 454 reads only (green), and all reads including Illumina and 454.

7.2 Per-sample sensitivity estimates

We compared Exon Pilot SNP calls in samples NA12878 and NA19240 to the variants called in the high-coverage whole-genome Trio Pilot data from the same samples, and found that the missed variant rates (FNR) were 3.3% and 4.4%, respectively. We also compared the Exon Pilot SNPs in the target regions of samples NA12878 and NA19240 to the variants called in the Complete Genomics whole-genome sequence of the same samples. The FNR of the Exon Pilot SNP calls for the two samples was 5.3% and 5.0%, respectively. The FNR for the Complete Genomics variant calls was estimated independently from the fraction of confirmed *de novo* germline mutations (data not shown), as 4.1% and 7.9% for the two samples, respectively. These numbers place the FNR estimates between 5.5% and 9.4% for NA12878, and between 5.0% and 12.9% for NA19240.

**8. Heterozygosity estimates of Exon and Low Coverage Pilots**

Heterozygosity estimates were carried out according to the methods reported in the main text (**Methods**). Detailed results are given in **Table S9** below.

a)

| Dataset | Sites | #Sites | YRI | LWK | CHB | CHD | JPT | CEU | TSI |
| --- | --- | --- | --- | --- | --- | --- | --- | --- | --- |
| Exon Pilot | All | 1315794 | 4.42 | 4.52 | 3.34 | 3.35 | 3.26 | 3.54 | 3.50 |
|  | 4-fold | 210575 | 9.24 | 9.16 | 6.60 | 6.63 | 6.43 | 7.12 | 7.04 |
|  | 3-fold | 20990 | 5.01 | 5.41 | 4.24 | 4.39 | 4.60 | 3.59 | 3.59 |
|  | 2-fold | 257486 | 6.04 | 6.16 | 4.44 | 4.42 | 4.37 | 4.74 | 4.68 |
|  | Nonsyn | 854682 | 2.74 | 2.86 | 2.19 | 2.21 | 2.12 | 2.31 | 2.29 |
| LC Pilot | Exon Pilot sites | 1293677 | 4.33 | N/A | 3.38 | N/A | 3.38 | 3.53 | N/A |
|  | All | 2258231293 | 9.39 | N/A | 6.90 | N/A | 6.90 | 7.49 | N/A |
|  | 4-fold | 5273705 | 9.08 | N/A | 6.84 | N/A | 6.84 | 7.26 | N/A |
|  | 3-fold | 466460 | 6.10 | N/A | 4.48 | N/A | 4.48 | 4.84 | N/A |
|  | 2-fold | 6107388 | 6.24 | N/A | 4.68 | N/A | 4.68 | 5.01 | N/A |
|  | Nonsyn | 20579051 | 3.36 | N/A | 2.74 | N/A | 2.74 | 2.74 | N/A |
| ENCODE | All |  | 2.70 | 2.78 | 1.85 | 1.94 | 1.86 | 1.89 | 1.85 |

b)

| Dataset | Sites | #Sites | YRI | LWK | CHB | CHD | JPT | CEU | TSI |
| --- | --- | --- | --- | --- | --- | --- | --- | --- | --- |
| Exon Pilot | All | 1315794 | 1.00 | 1.00 | 1.00 | 1.00 | 1.00 | 1.00 | 1.00 |
|  | 4-fold | 210575 | 2.09 | 2.02 | 1.97 | 1.98 | 1.97 | 2.01 | 2.01 |
|  | 3-fold | 20990 | 1.13 | 1.20 | 1.27 | 1.31 | 1.41 | 1.01 | 1.02 |
|  | 2-fold | 257486 | 1.36 | 1.36 | 1.33 | 1.32 | 1.34 | 1.33 | 1.34 |
|  | Nonsyn | 854682 | 0.61 | 0.63 | 0.65 | 0.66 | 0.65 | 0.65 | 0.65 |
| LC Pilot | Exon Pilot sites | 1293677 | 0.98 | N/A | 1.01 | N/A | 1.03 | 1.00 | N/A |
|  | All | 2258231293 | 2.12 | N/A | 2.07 | N/A | 2.11 | 2.12 | N/A |
|  | 4-fold | 5273705 | 2.05 | N/A | 2.04 | N/A | 2.10 | 2.05 | N/A |
|  | 3-fold | 466460 | 1.38 | N/A | 1.34 | N/A | 1.37 | 1.37 | N/A |
|  | 2-fold | 6107388 | 1.41 | N/A | 1.40 | N/A | 1.43 | 1.42 | N/A |
|  | Nonsyn | 20579051 | 0.76 | N/A | 0.82 | N/A | 0.84 | 0.77 | N/A |
| ENCODE | All |  | 0.61 | 0.62 | 0.55 | 0.58 | 0.57 | 0.53 | 0.53 |

c)

| Dataset | Sites | #Sites | YRI | LWK | CHB | CHD | JPT | CEU | TSI |
| --- | --- | --- | --- | --- | --- | --- | --- | --- | --- |
| Exon Pilot | All | 1315794 | 1.00 | 1.02 | 0.75 | 0.76 | 0.74 | 0.80 | 0.79 |
|  | 4-fold | 210575 | 1.00 | 0.99 | 0.71 | 0.71 | 0.70 | 0.77 | 0.76 |
|  | 3-fold | 20990 | 1.00 | 1.08 | 0.85 | 0.88 | 0.92 | 0.72 | 0.72 |
|  | 2-fold | 257486 | 1.00 | 1.02 | 0.74 | 0.73 | 0.72 | 0.78 | 0.77 |
|  | Nonsyn | 854682 | 1.00 | 1.04 | 0.80 | 0.81 | 0.77 | 0.84 | 0.84 |
| LC Pilot | Exon Pilot sites | 1293677 | 1.00 | N/A | 0.78 | N/A | 0.78 | 0.81 | N/A |
|  | All | 2258231293 | 1.00 | N/A | 0.73 | N/A | 0.73 | 0.79 | N/A |
|  | 4-fold | 5273705 | 1.00 | N/A | 0.75 | N/A | 0.75 | 0.79 | N/A |
|  | 3-fold | 466460 | 1.00 | N/A | 0.73 | N/A | 0.73 | 0.79 | N/A |
|  | 2-fold | 6107388 | 1.00 | N/A | 0.75 | N/A | 0.75 | 0.80 | N/A |
|  | Nonsyn | 20579051 | 1.00 | N/A | 0.81 | N/A | 0.81 | 0.82 | N/A |
| ENCODE | All |  | 1.00 | 1.03 | 0.68 | 0.72 | 0.69 | 0.70 | 0.68 |

**Table S9:** Per-base heterozygosity estimates of a) Exon, Low Coverage, and ENCODE b) normalized with respect to Exon Pilot values c) normalized with respect to YRI values. All values are in unit 10E-4.

**9. Allele sharing among populations**

To minimize possible biases resulting from fluctuations in coverage within and between populations, we restricted the analysis to samples with mean coverage of at least 15x and that were not otherwise identified as having high mismatch rate (Supplementary Section 3.2) This led to discarding 15 samples from CHB (NA18524, NA18529, NA18530, NA18531, NA18543, NA18557, NA18599, NA18615, NA18620, NA18627, NA18628, NA18635, NA18642, NA18749, NA18773), 16 samples from CEU (NA06985, NA06994, NA11840, NA11995, NA12004, NA12006, NA12156, NA12414, NA12763, NA12815, NA12829, NA12842, NA12872, NA12873, NA12874, NA12889), 21 samples from JPT (NA18948, NA18957, NA18961, NA18964, NA18969, NA18979, NA18981, NA18985, NA18988, NA18989, NA18994, NA19006, NA19054, NA19062, NA19065, NA19068, NA19077, NA19090, NA19554, NA19559, NA19562), 18 samples from YRI (NA18489, NA18499, NA18504, NA18516, NA18522, NA18865, NA18870, NA18871, NA18917, NA19102, NA19116, NA19137, NA19141, NA19181, NA19201, NA19207, NA19210, NA19220), 12 samples from TSI (NA20511, NA20512, NA20518, NA20519, NA20524, NA20527, NA20528, NA20529, NA20530, NA20540, NA20587, NA20763), 5 samples from CHD (NA17966, NA18109, NA18112, NA18147, NA18670), and 9 samples from LWK (NA19046, NA19307, NA19310, NA19312, NA19317, NA19321, NA19441, NA19453, NA19456).

Additionally, we only considered sites that were deemed to be "callable" in all the populations based on at least 10x coverage in at least 50 samples in all 7 panels, and 15x coverage for genotypes in populations that contained the non-reference allele.  The mean number of sites for which at least that passed all filters and contained at least two minor alleles after down-sampling was 2368 for coding and 1744 for noncoding.
