# Supplementary material for: The functional spectrum of low-frequency coding variation
Source: Genome Biol. 2011 Sep 14;12(9):R84. doi: 10.1186/gb-2011-12-9-r84 (PMC3308047; doi:10.1186/gb-2011-12-9-r84)
Supplement: Additional file 2 — 1000 Genomes Project members. List of the member of the pilot phase of the 1000 Genomes Project. [file gb-2011-12-9-r84-S2.DOC]

The 1000 Genomes Project Consortium

Wellcome Trust Sanger Institute, Wellcome Trust Genome Campus, Cambridge CB10 1SA, UK.

Richard M. Durbin, John Burton, David M. Carter, Carol Churcher, Alison Coffey, Anthony Cox, Aarno Palotie, Michael Quail, Tom Skelly, James Stalker, Harold P. Swerdlow, Daniel Turner, Qasim Ayub, Senduran Balasubramaniam, Jeffrey C. Barrett, Yuan Chen, Donald F. Conrad, Petr Danecek, Min Hu, Ni Huang, Matt E. Hurles, Luke Jostins, Thomas M. Keane, Si Quang Le, Sarah Lindsay, Quan Long, Daniel G. MacArthur, Leopold Parts, Chris Tyler-Smith, Klaudia Walter, Yali Xue, Yujun Zhang, Allison Coffey & Carol Scott

The Broad Institute of MIT and Harvard, 7 Cambridge Center, Cambridge, Massachusetts 02142, USA.

Stacey B. Gabriel, Eric S. Lander, Eric S. Lander (Principal Investigator), David Altshuler, Lauren Ambrogio, Toby Bloom, Kristian Cibulskis, Tim J. Fennell, Stacey B. Gabriel (Co-Chair), David B. Jaffe, Erica Shefler, Carrie L. Sougnez, Mark J. Daly (Principal Investigator), Mark A. DePristo (Project Leader), Aaron D. Ball, Eric Banks, Kiran V. Garimella, Sharon R. Grossman, Robert E. Handsaker, Matt Hanna, Chris Hartl, Andrew M. Kernytsky, Joshua M. Korn, Heng Li, Jared R. Maguire, Steven A. McCarroll, Aaron McKenna, James C. Nemesh, Anthony A. Philippakis, Ryan E. Poplin, Manuel A. Rivas, Pardis C. Sabeti, Stephen F. Schaffner, Ilya A. Shlyakhter, Mark A. DePristo & Jane Wilkinson

Center for Human Genetic Research, Massachusetts General Hospital, Boston, Massachusetts 02114, USA.

David Altshuler

Department of Genetics, Harvard Medical School, Cambridge, Massachusetts 02115, USA.

David Altshuler & Steven A. McCarroll

Center for Statistical Genetics and Biostatistics, University of Michigan, Ann Arbor, Michigan 48109, USA.

Yun Li (Project Leader), Paul Anderson, Tom Blackwell, Wei Chen, Jun Ding, Hyun Min Kang, Carlo Sidore, Matthew Snyder, Xiaowei Zhan, Sebastian Z√∂llner & Gon√ßalo R. Abecasis

Illumina Cambridge Ltd, Chesterford Research Park, Little Chesterford, Nr Saffron Walden, Essex CB10 1XL, UK.

David R. Bentley, Niall Gormley, Sean Humphray, Zoya Kingsbury, Paula Kokko-Gonzales, Jennifer Stone, R. Keira Cheetham, Tony Cox, Michael Eberle, Terena James, Scott Kahn & Lisa Murray

McKusick-Nathans Institute of Genetic Medicine, Johns Hopkins University School of Medicine, Baltimore, Maryland 21205, USA.

Aravinda Chakravarti

Center for Comparative and Population Genomics, Cornell University, Ithaca, New York 14850, USA.

Andrew G. Clark & Jeremiah Degenhardt

US National Institutes of Health, 1 Center Drive, Bethesda, Maryland 20892, USA.

Francis S. Collins

Life Technologies, Foster City, California 94404, USA.

Francisco M. De La Vega, Fiona C. L. Hyland, Onur Sakarya & Yongming A. Sun

Wellcome Trust Centre for Human Genetics, Roosevelt Drive, Oxford OX3 7BN, UK.

Peter Donnelly, Gil A. McVean, Adam Auton, Zamin Iqbal, Gerton Lunter, Jonathan L. Marchini & Simon Myers

Pall Corporation, 25 Harbor Park Drive, Port Washington, New York 11050, USA.

Michael Egholm

European Bioinformatics Institute, Wellcome Trust Genome Campus, Cambridge CB10 1SD, UK.

Paul Flicek, Laura Clarke (Project Leader), Fiona Cunningham, Javier Herrero, Stephen Keenen, Eugene Kulesha, Rasko Leinonen, William M. McLaren, Rajesh Radhakrishnan, Richard E. Smith, Vadim Zalunin & Xiangqun Zheng-Bradley

Human Genome Sequencing Center, Baylor College of Medicine, 1 Baylor Plaza, Houston, Texas 77030, USA.

Richard A. Gibbs, David Deiros, Mike Metzker, Donna Muzny, Jeff Reid, David Wheeler, Matthew Bainbridge, Danny Challis, Aniko Sabo, Fuli Yu, Jin Yu, Cristian Coafra, Huyen Dinh, Christie Kovar, Sandy Lee & Lynne Nazareth

Centre of Genomics and Policy, McGill University, Montr√©al, Qu√©bec H3A 1A4, Canada.

Bartha M. Knoppers

Max Planck Institute for Molecular Genetics, D-14195 Berlin-Dahlem, Germany.

Hans Lehrach, Ralf Sudbrak (Project Leader), Tatiana A. Borodina, Alexey N. Davydov, Peter Marquardt, Florian Mertes, Wilfiried Nietfeld, Aleksey V. Soldatov, Bernd Timmermann, Marius Tolzmann, Marcus W. Albrecht, Vyacheslav S. Amstislavskiy, Ralf Herwig & Dimitri V. Parkhomchuk

The Genome Center, Washington University School of Medicine, St Louis, Missouri 63108, USA.

Elaine R. Mardis, Richard K. Wilson, David Dooling, Lucinda Fulton, Robert Fulton, George Weinstock, Ken Chen, Asif Chinwalla, Li Ding, Daniel C. Koboldt, Mike D. McLellan, John W. Wallis, Michael C. Wendl & Qunyuan Zhang

Department of Statistics, University of Oxford, Oxford OX1 3TG, UK.

Jonathan L. Marchini, Loukas Moutsianas, Simon Myers, Afidalina Tumian & Gil A. McVean

Department of Genome Sciences, University of Washington School of Medicine, Seattle, Washington 98195, USA.

Deborah A. Nickerson, Gozde Aksay & Jeffrey M. Kidd

Wellcome Trust, Gibbs Building, 215 Euston Road, London NW1 2BE, UK.

Alan J. Schafer & Audrey Duncanson

US National Institutes of Health, National Center for Biotechnology Information, 45 Center Drive, Bethesda, Maryland 20892, USA.

Stephen T. Sherry, Richa Agarwala, Hoda M. Khouri, Aleksandr O. Morgulis, Justin E. Paschall, Lon D. Phan, Kirill E. Rotmistrovsky, Robert D. Sanders, Martin F. Shumway & Chunlin Xiao

BGI-Shenzhen, Shenzhen 518083, China.

Jun Wang, Min Jian, Guoqing Li, Ruiqiang Li, Huiqing Liang, Geng Tian, Bo Wang, Jian Wang, Wei Wang, Huanming Yang, Xiuqing Zhang, Huisong Zheng, Jun Wang (Principal Investigator), Xiaodong Fang, Xiaosen Guo, Yingrui Li, Ruibang Luo, Shuaishuai Tai, Honglong Wu, Hancheng Zheng, Xiaole Zheng, Yan Zhou, Taosha Li & Yeyang Su

Department of Biology, University of Copenhagen 2200, Denmark.

Jun Wang & Ruiqiang Li

Life Technologies, Beverly, Massachusetts 01915, USA.

Kevin J. McKernan (Principal Investigator), Gina L. Costa, Jeffry K. Ichikawa, Clarence C. Lee, Yutao Fu, Jonathan M. Manning, Stephen F. McLaughlin, Heather E. Peckham & Eric F. Tsung

Deep Sequencing Group, Biotechnology Center TU Dresden, Tatzberg 47/49, 01307 Dresden, Germany.

Andreas Dahl

Institute of Clinical Molecular Biology, Christian-Albrechts-University Kiel, Kiel 24105, Germany.

Philip Rosenstiel & Stefan Schreiber

Roche Applied Science, 20 Commercial Street, Branford, Connecticut 06405, USA.

Jason Affourtit, Dana Ashworth, Said Attiya, Melissa Bachorski, Eli Buglione, Adam Burke, Amanda Caprio, Christopher Celone, Shauna Clark, David Conners, Brian Desany, Lisa Gu, Lorri Guccione, Kalvin Kao, Andrew Kebbel, Jennifer Knowlton, Matthew Labrecque, Louise McDade, Craig Mealmaker, Melissa Minderman, Anne Nawrocki, Faheem Niazi, Kristen Pareja, Ravi Ramenani, David Riches, Wanmin Song, Cynthia Turcotte, Shally Wang, James Knight & Roger Winer

Department of Medical Genetics, Institute of Molecular Medicine (FIMM) of the

University of Helsinki and Helsinki University Hospital, Helsinki 00290, Finland.

Aarno Palotie

Agilent Technologies Inc., Santa Clara, California 95051, USA.

Anniek De Witte & Shane Giles

Department of Biology, Boston College, Chestnut Hill, Massachusetts 02467, USA.

Gabor T. Marth (Principal Investigator), Erik P. Garrison, Amit Indap, Deniz Kural, Wan-Ping Lee, Wen Fung Leong, Chip Stewart, Alistair N. Ward & Jiantao Wu

US National Institutes of Health, National Institute of Environmental Health Sciences, 111 T W Alexander Drive, Research Triangle Park, North Carolina 27709, USA.

Weichun Huang

Department of Biochemistry and Molecular Genetics, University of Virginia School of Medicine, Charlottesville, Virginia 22908, USA.

Aaron R. Quinlan

Illumina, San Diego, California 92121, USA.

Michael P. Stromberg

Department of Pathology, Brigham and Women‚Äôs Hospital and Harvard Medical School, Boston, Massachusetts 02115, USA.

Charles Lee (Principal Investigator), Ryan E. Mills, Xinghua Shi & David Altshuler

Department of Medicine, Division of Medical Genetics, University of Washington, Seattle, Washington 98195, USA.

Brian L. Browning

Center for Systems Biology, Department of Organismic and Evolutionary Biology, Harvard University, Cambridge, Massachusetts 02138, USA.

Sharon R. Grossman, Pardis C. Sabeti & Ilya A. Shlyakhter

Department of Epidemiology, Harvard School of Public Health, Boston, Massachusetts 02115, USA.

Alkes Price

Institute of Medical Genetics, Cardiff University, Heath Park, Cardiff CF14 4XN, UK.

David N. Cooper (Principal Investigator), Edward V. Ball, Matthew Mort, Andrew D. Phillips & Peter D. Stenson

Departments of Psychiatry and Cellular and Molecular Medicine, University of California San Diego, 9500 Gilman Drive, La Jolla, California 92093, USA.

Jonathan Sebat (Principal Investigator)

Seaver Autism Center and Department of Psychiatry, Mount Sinai School of Medicine, New York, New York 10029, USA.

Vladimir Makarov & Seungtai C. Yoon

Department of Epidemiology and Population Health, Albert Einstein College of Medicine, Bronx, New York 10461, USA.

Kenny Ye

Department of Genetics and Genomic Sciences, Mount Sinai School of Medicine, New York, New York 10029, USA.

Department of Genetics, Stanford University, Stanford, California 94305, USA.

Carlos D. Bustamante (Co-Principal Investigator), Michael Snyder (Co-Principal Investigator), Fabian Grubert, Hugo Y. K. Lam, Alexander E. Urban, Mark Kaganovich, Jeffrey M. Kidd & Simon Gravel

Department of Molecular and Cellular Biology, University of Arizona, Tucson, Arizona 85721, USA.

European Molecular Biology Laboratory, Genome Biology Research Unit, Meyerhofstrasse 1, Heidelberg 69117, Germany.

Adrian M. St√ºtz & Jan O. Korbel

Molecular Epidemiology Section, Medical Statistics and Bioinformatics, Leiden University Medical Center, 2333 ZA, The Netherlands.

Kai Ye

Department of Biological Sciences, Louisiana State University, Baton Rouge, Louisiana 70803, USA.

Mark A. Batzer (Principal Investigator), Miriam K. Konkel & Jerilyn A. Walker

The Translational Genomics Research Institute, 445 N Fifth Street, Phoenix, Arizona 85004, USA.

David W. Craig (Principal Investigator), Steve M. Beckstrom-Sternberg, Alexis Christoforides, Ahmet A. Kurdoglu, John V. Pearson, Shripad A. Sinari & Waibhav D. Tembe

Center for Biomolecular Science and Engineering, University of California Santa Cruz, Santa Cruz, California 95064, USA.

David Haussler (Principal Investigator), Angie S. Hinrichs, Sol J. Katzman, Andrew Kern & Robert M. Kuhn

Department of Human Genetics and Howard Hughes Medical Institute, University of Chicago, Chicago, Illinois 60637, USA.

Molly Przeworski

Department of Bioengineering and Therapeutic Sciences, University of California San Francisco, San Francisco, California 94158, USA.

Ryan D. Hernandez

Department of Human Genetics, University of Chicago, Chicago, Illinois 60637, USA.

Bryan Howie, Joanna L. Kelley & S. Cord Melton

National Heart and Lung Institute, Imperial College London, London SW7 2, UK.

William O. Cookson & Miriam F. Moffatt

Centre Nationale de G√©notypage, Evry 91000, France.

Mark Lathrop

Departments of Epidemiology and Biostatistics, Harvard School of Public Health, Boston, Massachusetts 02115, USA.

Liming Liang

Department of Epidemiology, University of Texas MD Anderson Cancer Center, Houston, Texas 77030, USA.

Paul Scheet

Department of Pediatrics, Faculty of Medicine, University of Montr√©al, Ste. Justine Hospital Research Centre, Montr√©al, Qu√©bec H3T 1C5, Canada.

Philip Awadalla (Principal Investigator)

Department of Medicine, Centre Hospitalier de l‚ÄôUniversit√© de Montr√©al Research Center, Universit√© de Montr√©al, Montr√©al, Qu√©bec H2L 2W5, Canada.

Ferran Casals, Youssef Idaghdour, Jonathan Keebler, Eric A. Stone & Martine Zilversmit

Eccles Institute of Human Genetics, University of Utah School of Medicine, Salt Lake City, Utah 84112, USA.

Jinchuan Xing & Lynn Jorde

Department of Genome Sciences, University of Washington School of Medicine and Howard Hughes Medical Institute, Seattle, Washington 98195, USA.

Evan E. Eichler & Can Alkan

Department of Computer Science, Simon Fraser University, Burnaby, British Columbia V5A 1S6, Canada.

Iman Hajirasouliha & Fereydoun Hormozdiari

Department of Haematology, University of Cambridge and National Health Service Blood and Transplant, Cambridge CB2 1TN, UK.

Cornelis A. Albers

Department of Genetic Medicine and Development, University of Geneva Medical School, Geneva 1211, Switzerland.

Emmanouil T. Dermitzakis & Stephen B. Montgomery

Center for Genome Science, Korea National Institute of Health, 194, Tongil-Lo, Eunpyung-Gu, Seoul 122-701, Korea.

Hanjun Jin

Program in Computational Biology and Bioinformatics, Yale University, New Haven, Connecticut 06520, USA.

Mark B. Gerstein (Co-Principal Investigator), Alexej Abyzov, Lukas Habegger, Rajini Haraksingh, Justin Jee, Jing Leng & Xinmeng Jasmine Mu

Department of Computer Science, Yale University, New Haven, Connecticut 06520, USA.

Robert Bjornson, Jiang Du & Mark B. Gerstein (Principal Investigator)

Department of Molecular Biophysics and Biochemistry, Yale University, New Haven, Connecticut 06520, USA.

Suganthi Balasubramanian, Ekta Khurana & Zhengdong Zhang

Department of Psychiatry and Behavioral Studies, Stanford University, Stanford, California 94305, USA.

Alexander E. Urban

Coriell Institute, 403 Haddon Avenue, Camden, New Jersey 08103, USA.

Neda Gharani & Lorraine H. Toji

Centre for Health, Law and Emerging Technologies, University of Oxford, Oxford OX3 7LF, UK.

Jane S. Kaye

Genetic Alliance, 436 Essex Road, London N1 3QP, UK.

Alastair Kent

Center for Medical Ethics and Health Policy, Baylor College of Medicine, 1 Baylor Plaza, Houston, Texas 77030, USA.

Amy L. McGuire

Department of Medical History and Bioethics, University of Wisconsin‚ÄìMadison, Madison, Wisconsin 53706, USA.

Pilar N. Ossorio

US National Institutes of Health, Center for Research on Genomics and Global Health, 12 South Drive, Bethesda, Maryland 20892, USA.

Charles N. Rotimi

US National Institutes of Health, National Human Genome Research Institute, 5635 Fishers Lane, Bethesda, Maryland 20892, USA.

Lisa D. Brooks, Adam L. Felsenfeld, Jean E. McEwen, Nicholas C. Clemm, Mark S. Guyer & Jane L. Peterson

The George Washington University School of Medicine and Health Sciences, Washington DC 20037, USA.

Assya Abdallah

US Food and Drug Administration, 11400 Rockville Pike, Rockville, Maryland 20857, USA.

Christopher R. Juenger

US National Institutes of Health, National Human Genome Research Institute, 31 Center Drive, Bethesda, Maryland 20892, USA.

Eric D. Green

Department of Ecology and Evolutionary Biology, Rice University, Houston, Texas 77251, USA.

Reed A. Cartwright
